# Is monitoring of plasma 5-fluorouracil levels in metastatic / advanced colorectal cancer clinically effective? A systematic review

**DOI:** 10.1186/s12885-016-2581-x

**Published:** 2016-07-25

**Authors:** Karoline Freeman, Mark P. Saunders, Olalekan A. Uthman, Sian Taylor-Phillips, Martin Connock, Rachel Court, Tara Gurung, Paul Sutcliffe, Aileen Clarke

**Affiliations:** 1 Division of Health Sciences, Medical School, University of Warwick, Gibbet Hill Campus, Coventry, CV4 7AL UK; 2 The Christie, 550 Wilmslow Road, Manchester, M20 4BX UK

**Keywords:** 5-fluorourcil, Colorectal cancer, Pharmacokinetic monitoring, Dose algorithms

## Abstract

**Background:**

Pharmacokinetic guided dosing of 5-fluorouracil chemotherapies to bring plasma 5-fluorouracil into a desired therapeutic range may lead to fewer side effects and better patient outcomes. High performance liquid chromatography and a high throughput nanoparticle immunoassay (My5-FU) have been used in conjunction with treatment algorithms to guide dosing. The objective of this study was to assess accuracy, clinical effectiveness and safety of plasma 5-fluorouracil guided dose regimen(s) versus standard regimens based on body surface area in colorectal cancer.

**Methods:**

We undertook a systematic review. MEDLINE; MEDLINE In-Process & Other Non-Indexed Citations; EMBASE; Cochrane Library; Science Citation Index and Conference Proceedings (Web of Science); and NIHR Health Technology Assessment Programme were searched from inception to January 2014. We reviewed evidence on accuracy of My5-FU for estimating plasma 5-fluorouracil and on the clinical effectiveness of pharmacokinetic dosing compared to body surface area dosing. Estimates of individual patient data for overall survival and progression-free survival were reconstructed from published studies. Survival and adverse events data were synthesised and examined for consistency across studies.

**Results:**

My5-FU assays were found to be consistent with reference liquid chromatography tandem mass spectrometry. Comparative studies pointed to gains in overall survival and in progression-free survival with pharmacokinetic dosing, and were consistent across multiple studies.

**Conclusions:**

Although our analyses are encouraging, uncertainties remain because evidence is mainly from outmoded 5-fluorouracil regimens; a randomised controlled trial is urgently needed to investigate new dose adjustment methods in modern treatment regimens.

**Electronic supplementary material:**

The online version of this article (doi:10.1186/s12885-016-2581-x) contains supplementary material, which is available to authorized users.

## Background

Colorectal cancer is the third most common cancer in the Western world and is the second most common cancer-related cause of death in combined male and female populations in the UK [1], United States and Canada [2]. In 2010 in the UK there were just under 16,000 deaths from colorectal cancer [3, 4].

5-flourouracil is used in a variety of chemotherapy regimens for several cancers including colorectal cancer. According to NICE guideline CG131 [5], UK colorectal cancer patients may receive one of several 5-fluorouracil-based first and second line chemotherapies depending on side effects experienced and patient’s preference. These include 5-fluorouracil alone as an infusion, 5-fluorouracil + FA (folinic acid) often as a 2 day infusion called the de Gramont 5-fluorouracil + FA regimen, FOLFOX4 or FOLFOX6 regimens (5-fluorouracil plus oxaliplatin and FA administered over 46 h). Table 1 details the doses of chemotherapy drugs and mode of administration in the various treatment regimens. FOLFOX6 is the most commonly used regimen in the UK and Europe partly due to increased convenience for patients. These regimens may be administered every 2 weeks for up to 12 cycles. In standard practice the dose of 5-fluorouracil is calculated from patient body surface area (BSA).

**Table 1:** Chemotherapy treatment regimens including 5-fluorouracil for the treatment of colorectal cancer [60]

• 5-fluorouracil + FA: Fluorouracil + Folinic acid • FOLFOX4: (oxaliplatin (85 mg/m^2^), folinic acid (200 mg/m^2^), 5-5-fluorouracil (loading dose of (400 mg/m^2^) iv bolus, then (600 mg/m^2^) administered via ambulatory for a period of 22 h • FOLFOX6: (oxaliplatin (85–100 mg/m^2^), folinic acid (400 mg/m^2^), 5-5-fluorouracil (loading dose of (400 mg/m^2^) iv bolus, then (2,400–3,000 mg/m^2^) administered via ambulatory infusion for a period of 46 h	

*Abbreviations*: *FA* folinic acid, *iv* intra venous

5-fluorouracil associated severe side effects (e.g. diarrhoea, hand and foot syndrome, mucositis/stomatitis, neutropenia, anaemia, nausea/vomiting, cardio toxicity) or anticipated risk of toxicity may lead to dose reductions, treatment ‘holidays’ or to dose capping for fear of overdose [6]. Orally administered 5-fluorouracil prodrug Capecitabine offers additional therapeutic choice but is unlikely to replace 5-fluoruracil [7].

Plasma 5-fluorouracil concentrations vary greatly between individuals who have received “standard” dosage calculated from their body surface area; this is because clearance is largely dependent on dihydropyrimidine-dehydrogenase activity which varies between individuals [8–12]. Other enzymes with minor roles and varying activities between individuals include thymidylate synthetase and methylenetetrahydrofolate reductase. As a result some patients may receive doses which are too low to be fully effective, whereas others may experience toxicity because their circulating dose is too high. Adjusting 5-fluorouracil dose to bring plasma concentrations into an appropriate therapeutic range is a potentially effective strategy to counteract these contingencies.

In pharmacokinetic (PK) regimens the first cycle dose is based on patient body surface area while subsequent doses are calculated based on individuals’ blood 5-fluorouracil concentrations. A steady state plasma sample is taken (e.g. after 40 h of a 46 h infusion) and the 5-fluorouracil estimate is used to calculate the PK “area under the curve (AUC)” (AUC = mg * H /L; where mg/L is the steady state plasma 5-fluorouracil concentration and H is the total infusion time in hours). This, and subsequent area under the curve estimates are used to adjust the 5-fluorouracil dose before each subsequent infusion [8].

In clinical practice for pharmacokinetic regimens plasma 5-fluorouracil has been estimated by in-house high performance liquid chromatography (HPLC) [13] or by commercial immunoassay using venous samples. The My5-FU assay is a nanoparticle immunoassay [14, 15] that can be performed on automated clinical chemistry analysers present in standard clinical laboratories thereby allowing high throughput of samples. The estimate is used to adjust the 5-fluorouracil dose at the next infusion according to a pre-specified dosage algorithm.

In this systematic review we: (a) examine the accuracy My5-FU assays, and describe dose adjustment algorithms developed from 5-fluorouracil assays; (b) assess the evidence on the clinical effectiveness of plasma 5-fluorouracil guided dose regimen(s) versus standard BSA-guided regimens as first line treatments in advanced / metastatic colorectal cancer; and (c) assess the safety of plasma 5-fluorouracil guided dose regimen(s) versus standard BSA-guided regimen(s).

Advanced colorectal cancer is taken here to be colorectal cancer that at presentation or recurrence is either metastatic or so locally advanced that surgical resection is unlikely to be carried out with curative intent.

## Methods

### Protocol registration

The study methods were specified in advance and documented in a study protocol. The study protocol was registered with PROSPERO, registration number CRD42014007213.

### Eligibility criteria

The following inclusion criteria were used to identify potentially relevant studies:

- *Population*: colorectal cancer patients receiving 5-fluorouracil chemotherapy by continuous venous infusion.
- *Intervention*: pharmacokinetic monitoring of plasma 5-fluorouracil to guide dose regimen.
- *Comparator*: body surface area based dose regimen (comparative studies only).
- *Outcomes*: test accuracy, progression free survival, overall survival, adverse events.
- *Study design*: comparative studies (specified intervention versus comparator) or single arm studies describing pharmacokinetic monitoring of plasma 5-fluorouracil used to adjust patients’ dose regimen.

### Information sources and search

We developed a broad search to identify studies covering clinical effectiveness, algorithms and test accuracy relating to My5-FU and other relevant technologies (Additional file 1). We searched MEDLINE; MEDLINE In-Process & Other Non-Indexed Citations; EMBASE; Cochrane Library (including Cochrane Systematic Reviews, DARE, CENTRAL, NHS EED, and HTA databases); Science Citation Index and Conference Proceedings (Web of Science); NIHR Health Technology Assessment Programme; (International Prospective Register of Systematic Reviews) from inception to January 2014. The following trial databases were also searched: Current Controlled Trials; ClinicalTrials.gov; UKCRN Portfolio Database; WHO International Clinical Trials Registry Platform. We searched reference lists of all identified primary studies and systematic reviews.

### Study selection and data extraction

Two reviewers independently screened titles and abstracts of all identified records and assessed potentially relevant full texts for eligibility using pre-specified inclusion criteria. Discrepancies were resolved through discussion. Data were extracted by one reviewer, using a piloted data extraction form. A second reviewer checked extracted data with disagreements resolved by consensus or discussion with a third reviewer. We contacted authors for additional data on overall and progression-free survival. We obtained full texts of appropriate additional primary studies on the clinical effectiveness of standard dosing 5-fluorouracil regimens from a recent comprehensive systematic review [5] to validate the standard care arm in pharmacokinetic monitoring studies. We reconstructed Kaplan-Meier survival plots from multiple studies and derived estimates of individual patient data to develop parametric distributions to provide modelled estimates of median and mean survival.

### Risk of bias assessment

Two reviewers independently evaluated the methodological quality of eligible studies. Quality assessment was undertaken using the Downs and Black [16] checklist. Quality assessment of test accuracy studies was undertaken using an adapted QUADAS-2 checklist (available from authors on request).

### Data synthesis

In the absence of individual participant data (IPD), we used the method of Guyot et al. [17] to reconstruct estimates of IPD from published Kaplan-Meier plots for progression-free survival and overall survival to a) reproduce Kaplan- Meier plots and estimate restricted survival times and b) to model life time survival estimates in terms of progression free survival and overall survival. For this the x/y coordinates of the published Kaplan-Meier plot are first digitised ensuring that coordinates are recorded enclosing all steps in the plot. These, together with data for the number of events and risk table information if available, are then analysed with an iterative algorithm, written in R statistical software, to develop an estimate of the underlying IPD of the study population. Censoring is assumed to be uniform between risk table time points. The Guyot et al. procedure has been compared with alternative methods in a recent simulation study [18]. We estimated restricted mean survival time (RMST) using the AUC of the Kaplan-Meier plot and its 95 % lower and upper confidence intervals for the observed data. For comparative studies the RMST relates to the maximum observed period common to both study arms.

Life time survival outcomes were modelled with standard parametric models in Stata version 11 for Windows using the stgenreg package of Crowther and Lambert [19] that generates 95 % confidence intervals with the delta method. Mean survival times and 95 % confidence intervals were estimated from the AUC of the modelled curve, and also from the equations for mean survival published by Davies et al. [20]. Goodness of fit of parametric models was judged visually and according to information criteria (Akaike information criterion, Bayesian information criterion). The 95 % confidence intervals around proportions were estimated using the binomial distribution. Relative risks and associated 95 % confidence intervals were estimated using the “metan” package [21] in Stata.

Studies may appear to provide evidence of an advantage in OS and PFS for PK adjustment relative to BSA regimens because of poor performance of BSA arms used in the comparison or lack of consistency within pharmacokineticly adjusted (PKA) studies in general. To address these possibilities we investigated additional management studies drawn from a recent comprehensive systematic review (NICE Clinical Guidelines CG131) of randomised trials using 5-fluorouracil regimens as first line treatments for advanced and / or metastatic colorectal cancer [5].

## Results

### Study selection

The PRISMA flow diagram (Additional file 2) illustrates the search results. Following deduplication we sifted 2,565 unique records and included 203 records in the full text sift of potentially relevant records of which 180 were subsequently excluded using our pre-specified inclusion criteria. This resulted in the inclusion of 23 eligible studies. Nineteen clinical effectiveness studies (Additional file 3) [22–40] investigated pharmacokinetic dose adjustment, three studies [15, 41, 42] examined the accuracy of My5-FU assays and three studies [27, 35, 43] described dose adjustment algorithms of which two were also clinical effectiveness studies.

### Risk of bias assessment

None of the studies we investigated were of high quality, all had important drawbacks in design, methods, and key outcome coverage; these factors limit their validity and generalisability. In the studies of test accuracy there was a high risk of bias predominantly due to patient selection. The evidence from the single arm studies is weak as conclusions were mainly based on small study populations investigated as case series, which are generally of lower quality because selection bias cannot be assessed. The only RCT [38] did not report methods of randomisation. In the other two comparative studies [23, 40], the absence of randomisation means that true comparability between groups is inevitably compromised.

### Accuracy of the My5-FU assay against reference standard methods

Three studies investigating accuracy against reference standard methods were included (see Additional file 4) [15, 41, 42]. In each the My5-FU assay was compared with liquid chromatography-tandem mass spectrometry (LC-MS/MS) used as the reference test. Bland-Altman upper and lower limits of agreement were provided in only one study [41] which reported a mean bias towards a 7 % (95 % CIs: 5.5 to 8.5 %) higher value for My5-FU assays compared to LC-MS/MS, with 95 % of the differences between the two test between −18 and +30 %. Another study reported a 23 ng/mL mean bias towards a higher measurement for My5FU [15]. It was difficult to fully gauge the quality of these studies because of missing report details such as the frequency of excluded samples.

### Dose adjustment algorithms

Three algorithms were found [27, 35, 43]. Each was developed using a different 5-fluoruracil infusion regimen (8, 22 and 46 h infusions respectively). The Gamelin et al. [44] and Ychou et al. [35] algorithms were developed from analysis of plasma 5-fluorouracil estimates in a small group of patients (40 and 38 respectively) who received treatment cycles of increasing 5-fluoruracil dose until an upper limit plasma concentration was reached or toxicity experienced. By relating plasma concentration to clinical response the authors established a therapeutic target range for plasma 5-fluorouracil. The Gamelin algorithm target was 2000–3000 μg/litre corresponding to a PK area under the curve (20 to 24 mg*hours/litre). The third algorithm by Kaldate et al. [43] was derived from retrospective analysis of a PK database of My5-FU values and dose changes for colorectal cancer patients treated with a FOLFOX6 regimen. The authors performed linear regression analysis of change in PK area under the curve (mg*hours/litre) versus change in dose for 307 cycle-pairs in which a dose change was implemented. Kaldate et al. [43] proposed an optimal target plasma 5-fluorouracil of 435 to 652 μg/litre corresponding to a PK area under the curve (20 to 30 mg*hours/litre). Further details of these algorithms are presented in Additional file 5.

### Clinical effectiveness of pharmacokinetic adjusted dose regimen(s) in colorectal cancer patients

Nineteen studies investigated clinical effectiveness of dose adjusted regimens; they were disparate in design, population, treatments, and outcomes (Additional file 3). Of these only six (3 comparative [38–40] and 3 single arm [23, 27, 28]) reported time to event analyses that could be used to reconstruct individual patient data. The remaining 13 studies reported median progression-free survival or median overall survival or overall response rates. Adverse events were inconsistently reported. Overall response rates with PK monitoring ranged from 0 % to 69.7 %; statistical heterogeneity was considerable (*p* < 0.001; I^2^ 92.8 %) and together with clinical heterogeneity precluded pooling of response rates. Median progression-free survival and median overall survival ranged from 3.3 to 16 months and 9.6 to 28 months respectively; clinical and statistical heterogeneity and the lack of confidence intervals for values precluded pooling.

**Fig. 1:**
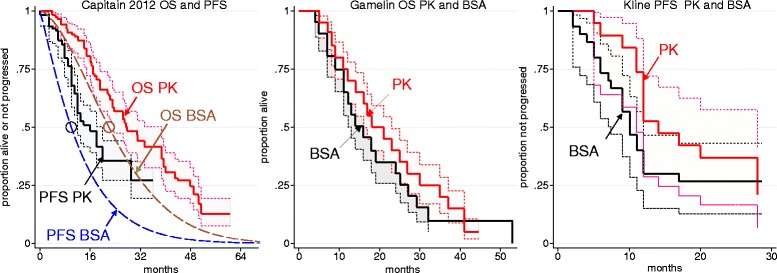
Reconstructed Kaplan Meier analyses (95 % CI) of the three dual arm studies. Capitain [39] used a FOLFOX6 regimen, Kline [40] FOLFOX6 or FOLFIRI regimens (the reported logrank test for pharmacokinetic versus body surface area was p 0.161), and Gamelin 2008 [38] an 5-fluorouracil + folinic acid (FO) regimen with 5-fluorouracil infused over 8 h (the reported logrank test for pharmacokinetic versus body surface area was p 0.08). The circular data points in the Capitain figure represent the reported medians for overall survival and progression-free survival in the body surface area arm and the dashed lines are Weibull fits using the same shape parameters as for Weibull fits to the pharmacokinetic arm

Kaplan-Meier plots of overall survival and progression free survival for the three comparative studies are shown in Fig. 1. The 8 h infusion of a 5FU + FO regimen by Gamelin et al. [38], resulted in a mean RMST of 17.95 months (95 % CI: 14.78–21.19) in the BSA arm and of 21.00 months (95 % CI: 17.71–24.14) in the PKA arm (observation period 60 months). Weibull parametric models for each arm yielded lifetime mean OS times of 19.64 months (95 % CI: 16.82–22.77) and 22.61 months (95 % CI: 19.65–25.85) for BSA and PKA arms respectively. For progression free survival (PFS) in Stage IV patients receiving the FOLFOX 6 or the FOLFIRI regimens of Kline et al. [40], the RMST was 13.00 months (95 % CI: 8.70–16.90) and 16.46 months (95%CI: 10.85–20.55) in the BSA and PKA arms, respectively (observation period 28 months). Weibull models yielded lifetime PFS values of 17.91 months (95 % CI: 11.40–31.48) and 19.57 months (95 % CI: 13.49–29.06), respectively.

Using the FOLFOX 6 regimen Capitain et al. [39] reported Kaplan-Meier plots of OS and PFS only for the PKA arm of the study; median OS and PFS survival without confidence intervals was reported for the comparator BSA arm. The RMST (observation period 60.5 months) for OS in the PKA arm was 31.13 months (95 % CI: 26.71–35.16) and the Weibull model yielded a mean lifetime OS value of 33.73 months (95 % CI: 29.21–38.93); the latter compares with a Weibull estimate of 24.5 months for the BSA arm estimated from the reported median survival and assuming proportional hazards with the PKA arm. The RMST (observation period 36 months) for PFS in the PKA arm was 18.52 months (95 % CI: 15.64–21.13) and the Weibull model yielded a lifetime PFS value of 25.07 months (95 % CI: 20.04–32.18); the latter compared with a Weibull estimate of 13.2 months for the BSA arm estimated from the median survival assuming proportional hazards with the PKA arm.

We identified three single arm 5-fluorouracil + folinic acid regimen PKA studies with published Kaplan-Maier plots. Gamelin et al. [27], and Gamelin et al. [28] used an 8 h infusion, and Capitain et al. [23] used a modified de Gramont regimen with a 46 h 5-fluorouracil infusion. Since Gamelin at al. [28] was the larger study by Gamelin et al. and may have included participants from the earlier report we only analysed Gamelin et al. [28] and Capitain et al. [23]. In Capitain et al. [23] the RMST for OS was 22.70 months (95 % CI: 17.37–28.30) (observation time 66.7 months) and the Weibull model yielded a lifetime mean OS value of 23.44 months (95 % CI: 18.70–29.33). In Gamelin et al. [28] the RMST for OS was 23.14 months (95 % CI: 19.60–26.71) (observation time 60.2 months) and the Weibull model yielded a lifetime mean OS value of 25.44 months (95 % CI: 21.8–29.44). Gamelin et al. [28] also reported PFS; the RMST for PFS was 11.5 months (95 % CI: 9.36–13.70) (observation time 34.9 months) and the Weibull model yielded a lifetime mean PFS value of 12.54 months (95 % CI: 10.35–15.23).

### Comparison of OS and PFS in BSA arms of comparative studies with RCTs using 5-fluorouracil regimens from NICE clinical guideline CG131

Four RCTs [45–48] provided Kaplan-Meier plots of OS for BSA arms of studies using 5-fluorouracil + folinate regimens and these can be compared with the BSA arm of the Gamelin et al. [38] RCT. Visual inspection revealed considerable similarity of these five KM plots (Figure S1, Additional file 6). The range of Weibull model estimates of mean lifetime OS for the RCT BSA arms (16.89 months, 95 % CI: 15.20–18.84 to 21.28, 95 % CI: 18.72–24.27) enclosed that for the Gamelin et al. [38] BSA arm (19.64 months; 95 % CI: 16.8–22.77) indicating consistency of the latter study with others in the public domain. Weibull models of PKA arms in studies using 5-fluorouracil + folinate regimens (Gamelin et al. [38] Gamelin et al. [28] and Capitain et al. [23]) delivered larger estimates of lifetime OS ranging from 22.6 to 25.44 months (data are summarised Additional file 7, Table S1).

Five RCTs provided Kaplan-Meier plots of OS for BSA arms of studies using FOLFOX 6 regimens [47, 49–52] (Additional file 6, Figure S1) and these can be compared with the BSA arm of the Capitain et al. [23] comparative study. A Weibull model for the Capitain BSA arm delivered an estimated 24.5 months mean lifetime OS whereas Weibull models for four of the CG131 yielded estimates of 18.1 (95 % CI: 16.48–19.89) to 22.98 (95 % CI: 18.20–29.10) months while for one CG131 study [47] the estimate was 28.19 (95 % CI: 23.10–34.80) months (Additional file 7, Table S1). It should be noted that a KM plot for the BSA arm was not provided in Capitain et al. [23]. The Weibull model of mean OS in the Capitain et al. [23] PKA arm was 33.73 months (95 % CI: 29.21–38.93) (Additional file 7, Table S1).

Three RCTs [45, 46, 48] reported very similar Kaplan-Meier plots for PFS of patients receiving a BSA 5-fluorouracil + folinate regimen (Additional file 6, Figure S2). Weibull model estimates of mean PFS were 7.65 (95 % CI: 6.58–8.90) [45], 6.97 (95 % CI: 6.24–7.77) [46], and 8.21 (95 % CI: 7.49–8.98) months [48]. These values are substantially less than the 12.54 (95 % CI: 10.35–15.23) months Weibull model estimate of mean PFS under a PKA 5-fluorouracil + folinate regimen based on Gamelin et al. [28] (Additional file 7, Table S2). Unfortunately there were no reports with KM plots for BSA arms in any PKA study that could be compared with these RCT study values.

Three RCTs [49, 50, 52] reported Kaplan-Meier plots for PFS of patients receiving FOLFOX 6 BSA regimens. By visual inspection these are similar to the plot for the BSA arm of the Kline et al. [40] comparative study (Additional file 6, Figure S2). Weibull model estimates of mean PFS for these RCTs were 10.66 (95 % CI: 9.01–12.59) [47], 11.41 (95 % CI: 9.90–13.14) [48], and 10.23 (95 % CI: 9.24–11.29) [50] months; that for Kline et al. [40] was 17.91 (95 % CI: 11.40–31.48) months, and for Capitain et al. [39] was 13.2 months. The corresponding modelled estimates for the PKA arms in Kline et al. [40] and Capitain et al. [39] were 19.57 (95 % CI: 13.49–29.06) and 25.1 months respectively (Additional file 7, Table S2).

Parametric model fits for the studies are summarised in Additional file 8.

### Adverse events of pharmacokinetic adjusted dose regimen(s) in colorectal cancer patients

**Fig. 2:**
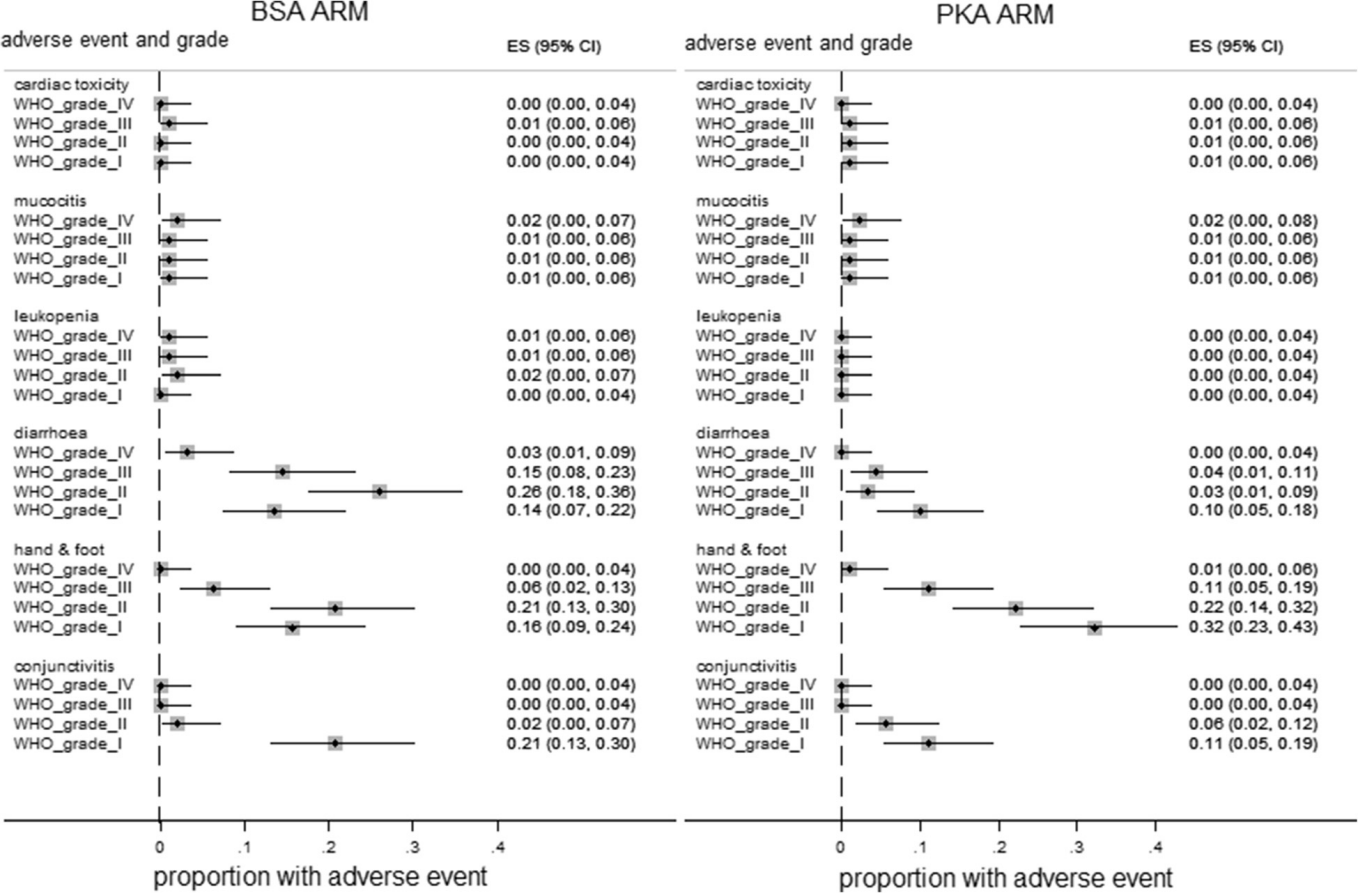
The incidence of various grades of adverse events in the BSA and PKA arms of Gamelin et al. [38]. The graph shows the proportion (95 % CI) of patients experiencing adverse events in the BSA and PKA arms during treatment with a 5-fluorouracil + folinic acid (FA) regimen in which 5-fluorouracil is infused over 8 h

The three studies comparing PK versus BSA based regimens [38–40] reported adverse events in different ways. With a FU + FA regimen Gamelin et al. [38] observed low incidence of all grades of cardiac toxicity, mucocitis, and leukopenia (≤2 % in both arms). Higher incidences of diarrhoea, hand and foot syndrome and WHO grades I and II conjunctivitis were found (Fig. 2). Leukopenia was less frequent in the PK arm (relative risk 0.36: 95 % CI: 0.01–8.61, for grades III and IV). All grades of diarrhoea were less frequent with the PKA regimen (relative risks for grades IV, III, II, and I were: 0.15, 95 % CI: CI: 0.01–2.91; 0.30, 95 % CI: CI: 0.01–0.89; 0.13, 95 % CI: 0.04–0.41; and 0.74, 95 % CI: 0.33–1.64 respectively; Additional file 9). All grades of hand and foot syndrome were more common in the PK arm but the frequency of grade IV hand and foot events was low (≤1 %). Patients in Capitain et al. [39] receiving the FOLFOX6 regimen experienced reduced incidence of diarrhoea (1.7 % versus 12 %) and mucocitis (0.8 % versus 15 %) and neutropenia (18 % versus 25 %) in scale categories III or IV of the National Cancer Institute’s Common Terminology Criteria with PK dosing; data for hand and foot syndrome was not reported. Patients in Kline et al. [40] received FOLFOX6 or FOLFIRI treatments. In this study incidence of toxic / adverse events was similar between PK and BSA based regimens, but onset was delayed with PK dosing.

## Discussion

### Summary and interpretation of findings

The dose of 5-fluorouracil-containing regimens given to cancer patients is widely based on the patient’s body surface area, but about 40 %–50 % of patients receiving 5-fluorouracil in this way may be under-dosed. Plasma 5-fluorouracil estimation in conjunction with dose adjustment algorithms might achieve more appropriate 5-fluorouracil dosing. We systematically reviewed the evidence on the clinical effectiveness and safety of pharmacokinetic dosing relative to dosing based on body surface area.

Although we identified 19 publications investigating clinical outcomes for pharmacokinetic 5-fluorouracil dose adjustment regimens only three studies compared PKA versus BSA, and only one of these was randomised. It is clear from the three comparative studies that there is an apparent advantage in PK monitoring for both progression-free survival and overall survival. Except for hand and foot syndrome, the particularly frequent and undesirable adverse events associated with 5-fluoruracil administration appear to be reduced and or delayed in these comparative studies so that taken in the round, these studies indicate that a PK dosing strategy is unlikely to be harmful and may lead to patient benefit, especially with regard to diarrhoea. This is supported by a recent community study reporting reduced grade 3 or 4 mucocitis and diarrhoea with PK dosing compared to historical controls [53].

So as to test the generalisability of overall survival and progression free survival reported in the studies of PKA dosing we examined the consistency of reported findings and compared the BSA arms of the comparative PKA versus BSA studies with BSA arms published in the literature (Additional files 6 and 7). It is clear that the BSA arms from the three PKA versus BSA comparative studies were well aligned with BSA arms from relevant studies [45–52] in both overall survival and progression free survival. Available time to event data for PK arms were consistent within their particular dose regimen (FU + FA or FOLFOX6) [23, 28, 38–40]. Estimates of median and mean survival times from well-fitting Weibull models of these studies indicate gains from PK monitoring (Additional file 7).

Unfortunately much of the evidence suggesting that PKA benefits overall and progression-free survival comes from 5-fluorouracil regimens that are now outmoded. These clinical studies have employed either HPLC or the My5-FU immunoassay procedure to estimate plasma 5-fluorouracil. Several studies suggest that a high correlation exists between My5-FU, HPLC and LC-MS/MS methods. Relative to reference assays with HPLC/tandem mass spectrometry the My5-FU immunoassay produced outlying estimates only at low plasma concentrations and with a degree of inaccuracy unlikely to lead to dangerous increases in dose when used in conjunction with suggested algorithms [27, 43]. On the available evidence, it seems unlikely that when used in conjunction with published dose adjustment algorithms these assays would result in dangerous overdosing.

### The place of My5-FU in clinical practice

Our review summarises the available evidence on PK dosing of 5-flourouracil in advanced colorectal cancer patients treated with 5-flourouracil and shows a link to favourable survival outcomes. My5-FU would seem to have a place in dose guidance to either reduce the 5-flourouracil dose and minimise toxicity or to increase the dose to prolong survival by generating high intra-tumour levels of 5-flourouracil that are effective in cancer treatment and prevent loss of response since a clear relationship between 5-flourouracil levels and response could be shown [27].

Currently, the 5-flourouracil dose given to individual patients to provide a certain response rate and overall survival is defined by different non-PK trials that do not allow dose increase. Therefore, within conventional efficacy, dose increases do not happen in clinical practice. However, if guided by My5-FU, increasing the dose would be a possibility for patients who have acceptable levels of side-effects.

It is important to keep in mind though that non-response to 5-flourouracil is not only related to dosage limitation. The main cause would be inherent resistance to 5-flourouracil rather than dose. Therefore dose adjustment is not going to prevent loss of response in all patients.

It is important that dose increases would be ruled by algorithms as well as clinical judgment. In 2011, Saam reported a US experience with My5-FU suggesting that physicians in practice made larger reductions than increases in 5-flourouracil doses [54]. And while Gamelin et al. [38] used an algorithm that allowed 50–70 % dose increases for some patients to reach the 5-flourouracil target range; it appears that physicians not bound to an adaptation protocol generally increased doses by only 10–20 %, illustrating a cautious attitude towards upward dose adjustment [54]. This might result in PK dose adjustment being less effective in clinical practice than in the research environment because different clinicians may apply dose increases more cautiously than in reported studies.

Under the current knowledge base it is hard to gauge where the My5-FU assay will fit into clinical practice. Successful pharmacokinetic dose adjustment using My5-FU in clinical practice will depend on a) accurate estimation of plasma 5-flourouracil, b) an appropriate algorithm for dose adaptation and c) an appropriate target plasma 5-flourouracil level. No currently available RCT or comparative study used the My5-FU assay for dose adjustment of 5-flourouracil containing chemotherapy regimens. As a result the current knowledge on the accurate estimation of plasma 5-flourouracil relies on comparisons with HPLC. The evidence on algorithms also comes from an indirect comparison with HPLC studies. Furthermore, the only algorithms currently available which have been validated in colorectal cancer patients are based on regimens no longer in clinical practice in the UK [36, 38] or are unavailable in the public domain [39]. It is unclear whether the survival gains can be generalised to other treatment regimens that may require alternative and as yet ill-defined adjustment algorithms [55]. Similarly it is unclear what the optimal plasma 5-flourouracil target level should be. Kaldate et al. [43] argue that newer extended infusion time regimens which are generally less toxic should use a wider target range of plasma 5-flourouracil levels with the upper limit increased to 30 mg*h/L than the initial target range of 20–24 mg*h/L established for the 8 h 5-flourouracil + folinic acid regimen [27]. However, no study was identified that made use of this algorithm. Most patients with CRC are nowadays treated with combination therapy the doses of which are defined by clinical trials. If a single agent fluoropyrimidine is needed, then capecitabine is often given. The most common 5-flourouracil combinations are with either irinotecan or oxaliplatin (FOLFIRI/FOLFOX). Even here, there are different types of FOLFOX. This complexity of treatment and modern treatment regimens are not reflected in the available trials on 5-flourouracil PK dosing.

The next step is therefore to evaluate the assay in combination therapy using standard of care regimens in clinical trials. When designing such trial several factors need to be considered: tumour type and combination of drugs, 5-flourouracil scheduling including oral fluoropyrimidines and the genetic makeup of colorectal cancer. Colorectal cancer has been divided into different molecular subtypes based on gene expression profiling. The success of PK dose adjustment is likely to vary by molecular subtype as an association between genotypes and outcomes could be shown [56–58]. With increasing use of genetic profiling, certain cohorts may be defined who would benefit more from PK testing. Understanding molecular biomarkers for predicting 5-flourouracil response could therefore aid appropriate use of PK dose adjustment.

In summary, it is hard to know where the My5-FU assay fits into clinical practice without the results of PK trials using the best available treatment for a given tumour type using conventional or PK dosing.

### Strengths and limitations

The main strengths of our study include the rigorous and comprehensive systematic review methodology applied, the comprehensive approach to the available evidence reaching from a highly sensitive search strategy to inclusion of comparative as well as single arm studies of clinical effectiveness of BSA and PKA dosing, the quantitative assessment and modelling of survival outcomes in individual studies, and our effort to assess the consistency of results reported in PKA studies from the perspective of the broader literature. There are several limitations in our review, these stem partly from the fact that the evidence on PKA versus BSA dosing in treating colorectal cancer is weak in both quantity and quality, from the fact that much of the evidence derives from outmoded treatment regimens, from the necessity of reconstructing individual patient data, and from the possibility that included studies may suffer from selective outcome reporting.

### Cost-effectiveness of PK dosing

Goldstein et al. [59] constructed a simple multistate Markov model to assess the cost effectiveness of PKA versus BSA dosing in a FOLFOX regimen. Survival estimates for the PKA arm were based on Capitain et al. [39] and for the BSA arm on Tournigand et al. [49]. Incidence of adverse events was taken from Capitain et al. [39] for the PKA arm and from Höchster et al. [51] for the BSA arm, and cost estimates were based on US practice. The authors estimated that PKA delivered an extra 1.46 quality adjusted life years at an extra cost $ 37,173. The incremental cost effectiveness ratio of $22,695/QALY was robust in univariate and multivariate sensitivity analyses. The authors concluded that at a $50,000/QALY threshold PK FOLFOX is cost effective for metastatic colorectal cancer and that it should be further evaluated in comparative effectiveness studies.

## Conclusions

The analyses are encouraging but the caveats about quality and relevance of the available evidence dictate caution. In order to compare pharmacokinetic (My5-FU or other) 5-fluorouracil dose adjustment with BSA-based dosing, a randomised controlled trial is urgently needed which compares intervention and control patients receiving a currently relevant 5-fluorouracil regimen. A trial would need to be developed with FOLFOX for example, given in a conventional way or a 5-flourouracil-PKA defined manner, to see if this increases patients’ response rate and overall survival or reduces toxicity without influencing overall survival. Research needs include: a) RCT of pharmacokinetic versus BSA dosing in metastatic and adjuvant colorectal cancer to include recent developments in genetic profiling; b) Evaluation of the comparability of different methods of current and any newly introduced pharmacokinetic dose adjustment; c) Randomised assessment of different algorithms for adjusting 5-fluorouracil dosing; and d) Further research on the quality of life impact of adverse events experienced in 5-fluorouracil treatments which would benefit economic assessments.

## Abbreviations

AUC, area under the curve; BSA, body surface area; FA, folinic acid; FOLFIRI, irinotecan in combination with 5-fluorouracil and folinic acid; FOLFOX, oxaliplatin in combination with 5-fluorouracil and folinic acid; FU, 5-fluorouracil; HPLC, High-performance liquid chromatography; LC-MS, liquid chromatography-mass spectrometry; OS, overall survival; PFS, progression free survival; PK, pharmacokinetic; PKA, pharmacokineticly adjusted

## Additional files

Additional file 1: Search strategy. (PDF 120 kb) Additional file 2: PRISMA study flow diagram. (PDF 15 kb) Additional file 3: Characteristics of included pharmacokinetic dose adjustment studies. (PDF 123 kb) Additional file 4: Characteristics of studies testing if My5-FU is clinically equivalent to LC-MS/MS. (PDF 14 kb) Additional file 5: Dose adjustment algorithms based on plasma 5-fluorouracil measurement in colorectal cancer patients. (PDF 148 kb) Additional file 6: Consistency of [A] overall survival and [B] progression free survival in body surface area guided dose arms of studies. (PDF 124 kb) Additional file 7: Modelled median and mean overall survival in pharmacokinetic and body surface area arms in studies of 5-fluorouracil + FA and FOLFOX6. (PDF 105 kb) Additional file 8: Parametric models of overall survival and progression free survival in 5-fluorouracil + folinate and FOLFOX6 regimens. (PDF 182 kb) Additional file 9: Relative risk of adverse events in the RCT of Gamelin (PK versus BSA dose adjustment strategies). (PDF 8 kb)
