# Supplementary material for: Is monitoring of plasma 5-fluorouracil levels in metastatic / advanced colorectal cancer clinically effective? A systematic review
Source: BMC Cancer. 2016 Jul 25;16:523. doi: 10.1186/s12885-016-2581-x (PMC4960837; doi:10.1186/s12885-016-2581-x)
Supplement: Additional file 1: — Search strategy. (PDF 120 kb) [file 12885_2016_2581_MOESM1_ESM.pdf]

Additional file 1 Search strategy

Embase Classic+Embase 1947 to 2014 Week 01 (Ovid), searched on 07/01/2014

1	(my5-fu* or My5-FU* or "my5 fu*" or "my 5fu*" or "my 5 fu*").mp.	6
2	ondose.mp.	6
3	saladax.mp.	10
4	1 or 2 or 3	18
5	"myriad genetic*".mp.	125
6	exp immunoassay/	389934
7	(immunoassay* or (immun* adj2 assay*)).mp.	379025
8	6 or 7	474599
9	high performance liquid chromatography/	197019
10	"high performance liquid chromatography".tw.	80851
11	HPLC.tw.	130008
12	"high pressure liquid chromatography".tw.	11005
13	high speed liquid chromatography.tw.	264
14	9 or 10 or 11 or 12 or 13	256728
15	liquid chromatography/ and mass spectrometry/	22033
16	Liquid chromatography-mass spectrometry.tw.	9168
17	LC?MS*.tw.	866
18	HPLC?MS.tw.	42
19	15 or 16 or 17 or 18	27415
20	((pharmacokinetic* or PK) adj2 (dosage* or dose* or dosing or adjust* or adapt* or monitor* or select* or calculat* or guided)).mp.	29883

21	fluorouracil/pk	2869
22	fluorouracil/	105631
23	(fluorouracil* or 5-fluorouracil* or 5fluorouracil*).tw.	37057
24	(5-fu* or 5fu* or fu).tw.	31818
25	22 or 23 or 24	118732
26	exp drug dose/	417095
27	drug monitoring/ or drug clearance/	79769
28	((dose* or dosing or dosage* or fluorouracil* or 5-fluorouracil* or 5fluorouracil* or 5-fu* or 5fu*) adj2 (adjust* or adapt* or monitor* or select* or calculat* or intensi* or escalat* or modif* or reduc* or concentration* or level* or limit* or detect* or measur*)).tw.	160112
29	((drug* or blood or plasma) adj5 (monitor* or concentration* or level*) adj5 (fluorouracil* or 5-fluorouracil* or 5fluorouracil* or 5-fu* or 5fu* or fu)).tw.	652
30	("optimal drug therapy" or ("optimal drug" adj (dosage* or dose* or dosing))).tw.	338
31	26 or 27 or 28 or 29 or 30	604858
32	personalized medicine/ and exp chemotherapy/	865
33	((personal* or individual*) adj2 (chemotherap* or dosage* or dose* or dosing)).mp.	10000
34	32 or 33	10652
35	31 or 34	611253
36	5 and 25	5
37	5 and 35	5
38	36 or 37	8
39	8 and 25 and 35	251
40	((5-fu* or 5fu* or fu) adj "plasma assay*").mp.	2

41	21 and 35	1315
42	4 or 38 or 39 or 40 or 41	1565
43	14 and 25	1263
44	19 and 25	95
45	43 or 44	1331
46	35 and 45	496
47	20 and 25	319
48	42 or 46 or 47	2102

MEDLINE(R) 1946 to November Week 3 2013 (Ovid), searched on 07/01/2014

1	(my5-fu* or My5-FU* or "my5 fu*" or "my 5fu*" or "my 5 fu*").mp.	0
2	ondose.mp.	2
3	saladax.mp.	1
4	1 or 2 or 3	3
5	"myriad genetic*".mp.	92
6	exp Immunoassay/	453924
7	(immunoassay* or (immun* adj2 assay*)).mp.	248384
8	6 or 7	527480
9	Chromatography, High Pressure Liquid/	155449
10	"high performance liquid chromatography".tw.	65042
11	HPLC.tw.	91531
12	"high pressure liquid chromatography".tw.	9702
13	high speed liquid chromatography.tw.	156
14	9 or 10 or 11 or 12 or 13	209442
15	exp Chromatography, Liquid/ and exp Mass Spectrometry/	65715
16	Liquid chromatography-mass spectrometry.tw.	7763
17	LC?MS*.tw.	365
18	HPLC?MS.tw.	5
19	15 or 16 or 17 or 18	68737
20	((pharmacokinetic* or PK) adj2 (dosage* or dose* or dosing or adjust* or adapt* or monitor* or select* or calculat* or guided)).mp.	6441
21	exp Fluorouracil/pk	1533
22	exp Fluorouracil/	42066

23	(fluorouracil* or 5-fluorouracil* or 5fluorouracil*).tw.	29605
24	(5-fu* or 5fu* or fu).tw.	21573
25	22 or 23 or 24	55027
26	Dose-response Relationship, Drug/ or Drug Dosage Calculations/	356879
27	Drug Monitoring/ or Metabolic Clearance Rate/	36771
28	((dose* or dosing or dosage* or fluorouracil* or 5-fluorouracil* or 5fluorouracil* or 5-fu* or 5fu*) adj2 (adjust* or adapt* or monitor* or select* or calculat* or intensi* or escalat* or modif* or reduc* or concentration* or level* or limit* or detect* or measur*)).tw.	111057
29	((drug* or blood or plasma) adj5 (monitor* or concentration* or level*) adj5 (fluorouracil* or 5-fluorouracil* or 5fluorouracil* or 5-fu* or 5fu* or fu)).tw.	549
30	("optimal drug therapy" or ("optimal drug" adj (dosage* or dose* or dosing))).tw.	231
31	26 or 27 or 28 or 29 or 30	476498
32	Individualized Medicine/	4498
33	((personal* or individual*) adj2 (chemotherap* or dosage* or dose* or dosing)).mp.	5704
34	32 or 33	10111
35	31 or 34	484057
36	5 and 25	3
37	5 and 35	4
38	36 or 37	5
39	8 and 25 and 35	172
40	((5-fu* or 5fu* or fu) adj "plasma assay*").mp.	1
41	21 and 35	773

42	4 or 38 or 39 or 40 or 41	942
43	14 and 25	857
44	19 and 25	138
45	43 or 44	904
46	35 and 45	319
47	20 and 25	92
48	42 or 46 or 47	1171

Medline In-Process & Other Non-Indexed Citations January 07, 2014 to November Week 3 2013 (Ovid), searched on 07/01/2014

1	(my5-fu* or My5-FU* or "my5 fu*" or "my 5fu*" or "my 5 fu*").mp.	1
2	ondose.mp.	0
3	saladax.mp.	1
4	1 or 2 or 3	1
5	"myriad genetic*".mp.	11
6	(immunoassay* or (immun* adj2 assay*)).mp.	9114
7	"high performance liquid chromatography".tw.	9130
8	HPLC.tw.	7931
9	"high pressure liquid chromatography".tw.	470
10	high speed liquid chromatography.tw.	25
11	7 or 8 or 9 or 10	14539
12	Liquid chromatography-mass spectrometry.tw.	872
13	LC?MS*.tw.	61
14	HPLC?MS.tw.	0
15	12 or 13 or 14	924
16	((pharmacokinetic* or PK) adj2 (dosage* or dose* or dosing or adjust* or adapt* or monitor* or select* or calculat* or guided)).mp.	365
17	(fluorouracil* or 5-fluorouracil* or 5fluorouracil*).tw.	1413
18	(5-fu* or 5fu* or fu).tw.	1243
19	17 or 18	2089
20	((dose* or dosing or dosage* or fluorouracil* or 5-fluorouracil* or 5fluorouracil* or 5-fu* or 5fu*) adj2 (adjust* or adapt* or monitor* or select*	6026

	or calculat* or intensi* or escalat* or modif* or reduc* or concentration* or level* or limit* or detect* or measur*)).tw.	
21	((drug* or blood or plasma) adj5 (monitor* or concentration* or level*) adj5 (fluorouracil* or 5-fluorouracil* or 5fluorouracil* or 5-fu* or 5fu* or fu)).tw.	19
22	("optimal drug therapy" or ("optimal drug" adj (dosage* or dose* or dosing))).tw.	19
23	20 or 21 or 22	6051
24	((personal* or individual*) adj2 (chemotherap* or dosage* or dose* or dosing)).mp.	306
25	23 or 24	6275
26	5 and 19	1
27	5 and 25	0
28	26 or 27	1
29	6 and 19 and 25	2
30	((5-fu* or 5fu* or fu) adj "plasma assay*").mp.	0
31	4 or 28 or 29 or 30	3
32	11 and 19	47
33	15 and 19	6
34	32 or 33	52
35	25 and 34	9
36	16 and 19	2
37	31 or 35 or 36	12

Cochrane Library (Wiley), searched on 17/01/2014

#1	("my5-fu" or My5-FU* or "my5 fu" or "my 5fu" or "my 5 fu"):ti,ab,kw	0
#2	ondose:ti,ab,kw	0
#3	saladax:ti,ab,kw	0
#4	#1 or #2 or #3	0
#5	(myriad next genetic*):ti,ab,kw	2
#6	[mh immunoassay]	4017
#7	(immunoassay* or (immun* near/2 assay*)):ti,ab,kw	4787
#8	#6 or #7	6316
#9	[mh ^"Chromatography, High Pressure Liquid"]	2376
#10	("high performance liquid chromatography"):ti,ab,kw	2356
#11	HPLC:ti,ab,kw	2450
#12	("high pressure liquid chromatography"):ti,ab,kw	388
#13	("high speed liquid chromatography"):ti,ab,kw	1
#14	#9 or #10 or #11 or #12 or #13	5409
#15	[mh "Chromatography, Liquid"]	2788
#16	[mh "Mass Spectrometry"]	1052
#17	#15 and #16	581
#18	("liquid chromatography-mass spectrometry"):ti,ab,kw	157
#19	("LC-MS" or LCMS* or "LC MS"):ti,ab,kw	443
#20	("HPLC-MS" or HPLCMS* or "HPLC MS"):ti,ab,kw	105
#21	#18 or #19 or #20	651
#22	((pharmacokinetic* or PK) near/2 (dosage* or dose* or dosing or adjust* or adapt* or monitor* or select* or calculat* or guided)):ti,ab,kw	2463
#23	[mh Fluorouracil/PK]	68

#24	[mh Fluorouracil]	3825
#25	(fluorouracil* or 5-fluorouracil* or 5fluorouracil*):ti,ab,kw	5908
#26	("5 fu" or 5fu* or fu):ti,ab,kw	2903
#27	#24 or #25 or #26	7097
#28	[mh "Dose-Response Relationship, Drug"]	24110
#29	[mh "Drug Dosage Calculations"]	66
#30	[mh ^"Drug Monitoring"]	1032
#31	[mh ^"Metabolic Clearance Rate"]	1544
#32	((dose* or dosing or dosage* or fluorouracil* or 5-fluorouracil* or 5fluorouracil* or "5-fu" or 5fu*) near/2 (adjust* or adapt* or monitor* or select* or calculat* or intensi* or escalat* or modif* or reduc* or concentration* or level* or limit* or detect* or measur*)):ti,ab,kw	14997
#33	((drug* or blood or plasma) near/5 (monitor* or concentration* or level*) near/5 (fluorouracil* or 5-fluorouracil* or 5fluorouracil* or "5-fu" or 5fu* or fu)):ti,ab,kw	47
#34	("optimal drug therapy" or ("optimal drug" next (dosage* or dose* or dosing))):ti,ab,kw	44
#35	#28 or #29 or #30 or #31 or #32 or #33 or #34	37921
#36	[mh ^"Individualized Medicine"]	78
#37	((personal* or individual*) near/2 (chemotherap* or dosage* or dose* or dosing)):ti,ab,kw	771
#38	#36 or #37	843
#39	#35 or #38	38431
#40	#5 and #27	1
#41	#5 and #39	0
#42	#40 or #41	1

#43	#8 and #27 and #39	5
#44	(("5-fu" or 5fu* or fu) next (plasma next assay*)):ti,ab,kw	0
#45	#23 and #39	45
#46	#4 or #42 or #43 or #44 or #45	51
#47	#14 and #27	29
#48	#21 and #27	4
#49	#47 or #48	33
#50	#39 and #49	17
#51	#22 and #27	15
#52	#46 or #50 or #51	67

All Results (67)

Cochrane Reviews (0)

Trials (65)

Methods Studies (0)

Technology Assessments (2)

Economic Evaluations (0)

Cochrane Groups (0)

SCI and SSCI via Web of Science searched on 09/01/2014

# 37	#31 OR #35 OR #36<br><br>Databases=SCI-EXPANDED, CPCI-S Timespan=All years	399
# 36	#16 AND #19<br><br>Databases=SCI-EXPANDED, CPCI-S Timespan=All years	156
# 35	#25 AND #34<br><br>Databases=SCI-EXPANDED, CPCI-S Timespan=All years	228

# 34	#32 OR #33<br><br>Databases=SCI-EXPANDED, CPCI-S Timespan=All years	731
# 33	#15 AND #19<br><br>Databases=SCI-EXPANDED, CPCI-S Timespan=All years	96
# 32	#11 AND #19<br><br>Databases=SCI-EXPANDED, CPCI-S Timespan=All years	680
# 31	#4 OR #28 OR #29 OR #30<br><br>Databases=SCI-EXPANDED, CPCI-S Timespan=All years	40
# 30	TS=((5-fu* OR 5fu* OR fu) NEAR/1 (plasma NEAR/1 assay*))<br><br>Databases=SCI-EXPANDED, CPCI-S Timespan=All years	2
# 29	#6 AND #19 AND #25<br><br>Databases=SCI-EXPANDED, CPCI-S Timespan=All years	34
# 28	#26 OR #27<br><br>Databases=SCI-EXPANDED, CPCI-S Timespan=All years	3
# 27	#5 AND #25<br><br>Databases=SCI-EXPANDED, CPCI-S Timespan=All years	2
# 26	#5 AND #19<br><br>Databases=SCI-EXPANDED, CPCI-S Timespan=All years	2
# 25	#23 OR #24<br><br>Databases=SCI-EXPANDED, CPCI-S Timespan=All years	131,943
# 24	TS=((personal* OR individual*) NEAR/2 (chemotherap* OR dosage* OR dose* OR dosing))<br><br>Databases=SCI-EXPANDED, CPCI-S Timespan=All years	6,959
# 23	#20 OR #21 OR #22	127,286

	Databases=SCI-EXPANDED, CPCI-S Timespan=All years	
# 22	TS=("optimal drug therapy" OR ("optimal drug" NEAR/1 (dosage* OR dose* OR dosing)))<br><br>Databases=SCI-EXPANDED, CPCI-S Timespan=All years	186
# 21	TS=((drug* OR blood OR plasma) NEAR/5 (monitor* OR concentration* OR level*) NEAR/5 (fluorouracil* OR 5-fluorouracil* OR 5fluorouracil* OR 5-fu* OR 5fu* OR fu))<br><br>Databases=SCI-EXPANDED, CPCI-S Timespan=All years	415
# 20	TS=((dose* OR dosing OR dosage* OR fluorouracil* OR 5-fluorouracil* OR 5fluorouracil* OR 5-fu* OR 5fu*) NEAR/2 (adjust* OR adapt* OR monitor* OR select* OR calculat* OR intensi* OR escalat* OR modif* OR reduc* OR concentration* OR level* OR limit* OR detect* OR measur*))<br><br>Databases=SCI-EXPANDED, CPCI-S Timespan=All years	127,025
# 19	#17 OR #18<br><br>Databases=SCI-EXPANDED, CPCI-S Timespan=All years	46,445
# 18	TS=(5-fu* OR 5fu* OR fu)<br><br>Databases=SCI-EXPANDED, CPCI-S Timespan=All years	22,299
# 17	TS=(fluorouracil* OR 5-fluorouracil* OR 5fluorouracil*)<br><br>Databases=SCI-EXPANDED, CPCI-S Timespan=All years	34,612
# 16	TS=((pharmacokinetic* OR PK) NEAR/2 (dosage* OR dose* OR dosing OR adjust* OR adapt* OR monitor* OR select* OR calculat* OR guided))<br><br>Databases=SCI-EXPANDED, CPCI-S Timespan=All years	11,242
# 15	#12 OR #13 OR #14<br><br>Databases=SCI-EXPANDED, CPCI-S Timespan=All years	38,936
# 14	TS=HPLC$MS*<br><br>Databases=SCI-EXPANDED, CPCI-S Timespan=All years	2

# 13	TS=LC$MS*<br><br>Databases=SCI-EXPANDED, CPCI-S Timespan=All years	44
# 12	TS=("liquid chromatography" NEAR/3 "mass spectrometry")<br><br>Databases=SCI-EXPANDED, CPCI-S Timespan=All years	38,898
# 11	#7 OR #8 OR #9 OR #10<br><br>Databases=SCI-EXPANDED, CPCI-S Timespan=All years	185,457
# 10	TS="high speed liquid chromatography"<br><br>Databases=SCI-EXPANDED, CPCI-S Timespan=All years	300
# 9	TS="high pressure liquid chromatography"<br><br>Databases=SCI-EXPANDED, CPCI-S Timespan=All years	7,726
# 8	TS=HPLC<br><br>Databases=SCI-EXPANDED, CPCI-S Timespan=All years	135,862
# 7	TS="high performance liquid chromatography"<br><br>Databases=SCI-EXPANDED, CPCI-S Timespan=All years	79,350
# 6	TS=(immunoassay* OR (immun* NEAR/2 assay*))<br><br>Databases=SCI-EXPANDED, CPCI-S Timespan=All years	158,922
# 5	TS=(myriad NEAR/1 genetic*)<br><br>Databases=SCI-EXPANDED, CPCI-S Timespan=All years	106
# 4	#1 OR #2 OR #3<br><br>Databases=SCI-EXPANDED, CPCI-S Timespan=All years	4
# 3	TS=saladax<br><br>Databases=SCI-EXPANDED, CPCI-S Timespan=All years	2
# 2	TS=ondose<br><br>Databases=SCI-EXPANDED, CPCI-S Timespan=All years	2

# 1	TS=((my5-fu*) OR (My5-FU*) OR "my5 fu" OR (my NEAR/1 5fu*) or "my 5 fu")<br><br>Databases=SCI-EXPANDED, CPCI-S Timespan=All years	1
