# Supplementary figures and images for: Is monitoring of plasma 5-fluorouracil levels in metastatic / advanced colorectal cancer clinically effective? A systematic review
Source: BMC Cancer. 2016 Jul 25;16:523. doi: 10.1186/s12885-016-2581-x (PMC4960837; doi:10.1186/s12885-016-2581-x)

**ADDITIONAL FILE 2. PRISMA Flow Diagram of studies included**

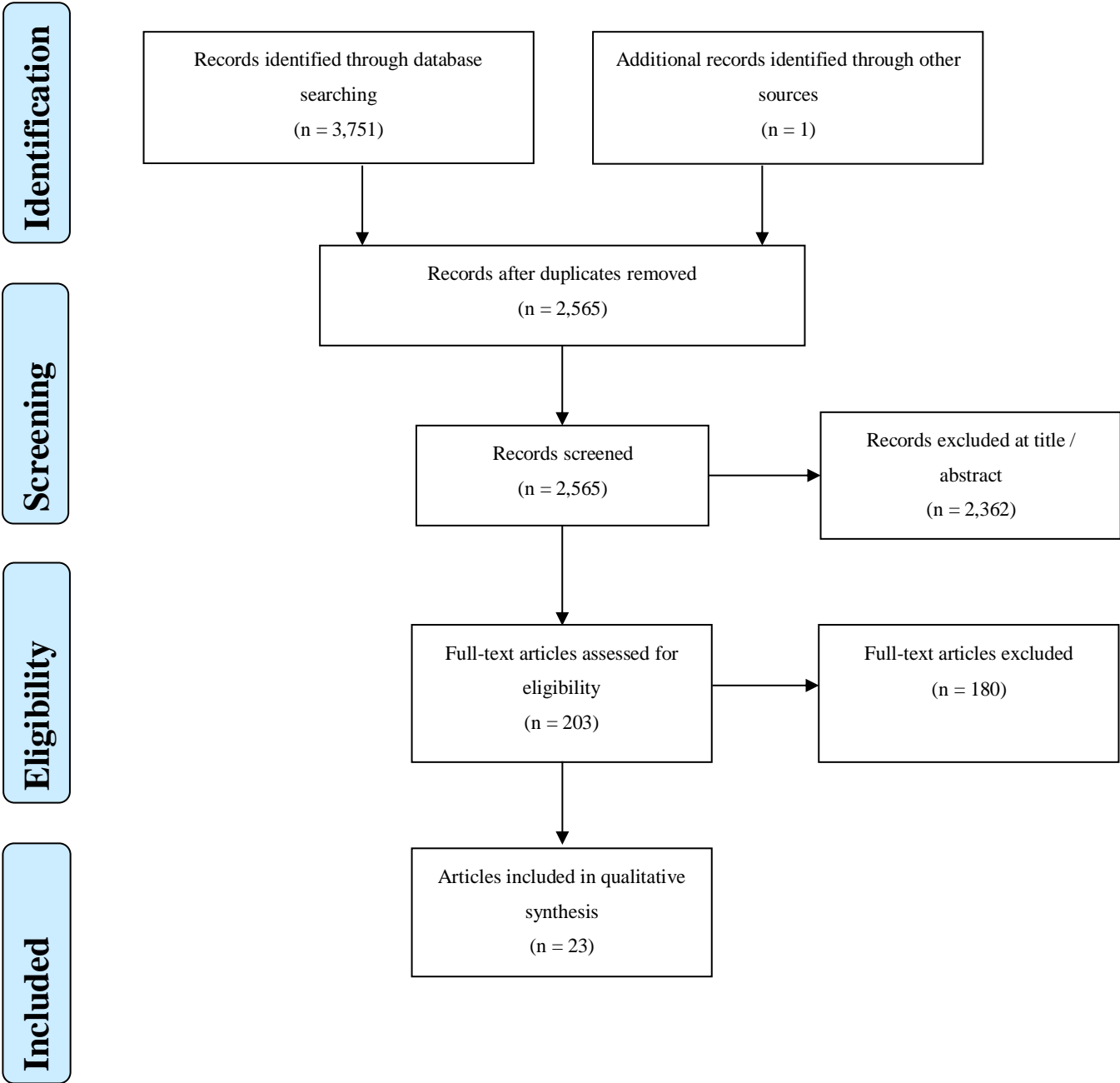

Supplement: Additional file 2: — PRISMA study flow diagram. (PDF 15 kb) [file 12885_2016_2581_MOESM2_ESM.pdf]
