# Supplementary material for: Is monitoring of plasma 5-fluorouracil levels in metastatic / advanced colorectal cancer clinically effective? A systematic review
Source: BMC Cancer. 2016 Jul 25;16:523. doi: 10.1186/s12885-016-2581-x (PMC4960837; doi:10.1186/s12885-016-2581-x)
Supplement: Additional file 3: — Characteristics of included pharmacokinetic dose adjustment studies. (PDF 123 kb) [file 12885_2016_2581_MOESM3_ESM.pdf]

ADDITIONAL FILE 3

Table 1. Characteristics of included pharmacokinetic dose adjustment studies

**First author, date, country.** ***Study design***	**Regimen**	**Algorithm** ***5-fluorouracil assay***	**N** pharmacokinetic ***N body surface area***	**5-fluorouracil related adverse events**	**Overall response rate** ***(%)***	**Overall survival median (months)** ***KM***	**Progression-free survival median (months)** ***KM***
**Studies with both pharmacokinetic and body surface area arms**							
Gamelin, 2008[35] France. *RCT*	5-fluorouracil (8h inf) + FA	Gamelin 1996[24] *HPLC*	104 *104*	Risk	pharmacokinetic 35/104 *(33.6)* body surface area 18/104 *(17.3)*	16 body surface area | 22 pharmacokinetic *Yes*	NR *No*
Capitain, 2012[36] France. *Retrospective case series + historical control*	FOLFOX6	Gamelin 1996[24] *HPLC*	118 *39*	Risk	pharmacokinetic 83/118 *(69.7)* body surface area18/39 *(46.6)*	22 body surface area | 28 pharmacokinetic *Yes**	10 body surface area | 16 pharmacokinetic *Yes**
Kline, 2014[37] USA. *Retrospective with two self-selected groups*	FOLFOX6 or FOLFIRI	Unclear *My5-FU*	19 *30*	Risk¶	NR	No *No*	10 body surface area | 14 pharmacokinetic *Yes*
**Studies with only a pharmacokinetic arm**							
Capitain, 2008[20] France. *Case series*	5-fluorouracil + FA (5-fluorouracil+LV, & modified de Gramont)	Gamelin 1996[24] *HPLC*	76	Risk §	25/76 *(32.9)*	20 *Yes*	3.28 *No*
Gamelin, 1996[24] France *Prospective case series (phase II study)*	5-fluorouracil (8h inf)	None *HPLC*	40	Risk	18/40 *(45)*	14 *Yes*	Unclear *Yes*
Gamelin, 1998[25] France *Prospective case series (multicentre phase II)*	5-fluorouracil (8h inf)	Gamelin 1996[24] *HPLC*	152	Counts	66/117 *(56.4)*	19 *Yes*	11 *Yes*
Boisdron-Celle, 2002[19] France. *Prospective case series*	5-fluorouracil + FA (+ platin post progression)	Gamelin 1996[24] *HPLC*	29	Counts §§	7/27 *(25.9)*	NR *No*	NR *No*
Cattel, 2003[21] Italy. *Prospective case series*	5-fluorouracil (14 day inf) + platin	None *HPLC*	13	No	7/13 *(53)*	9.6 *No*	7 *No*
Duffeur, 2010[22] France. *Retrospective database analysis*	De Gramont (LV 5-fluorouracil2)	Ychou 2003[33] *HPLC*	103	Risk	young 15/55 *(27)* elderly 17/48 *(35)*	young 18.7 elderly 13.4 *No*	NR *No*
Findlay, 1996[23] UK. *Case series*	5-fluorouracil (not specified)	None *HPLC*	19	Risk §	8/19 *(42)*	No *No*	NR *No*
Ho, 2011[26] China *Prospective case series*	5-fluorouracil (48h infusion) + FA	None *HPLC*	16	Counts	3/16 *(18.8)*	10.5 *No*	4.1 *No*
Jodrell, 2001[27] UK *Prospective case series and simulation study*	5-fluorouracil (protracted 1-26 weeks)	None *HPLC*	61	Risk §	16/61 *(26)*	11 *No*	NR *No*

First author, date, country. ***Study design***	**Regimen**	**Algorithm** ***5-fluorouracil assay***	**N** pharmacokinetic ***N body surface area***	**5-fluorouracil related adverse events**	**Overall response rate *(%)***	**Overall survival median (months)** ***KM***	**Progression-free survival median (months)** ***KM***
Kline, 2011[28] USA *Case series*	FOLFOX6 + Avastatin, FOLFOX6, FOLFIRI, FOLXOX4	Unclear *My5-FU*	21	NR	NR	NR *No*	NR *No*
Metzger, 1994[29] France *RCT**	5-fluorouracil (5-day infusion, flat or chronomodulated) + FA + platin	None *HPLC*	9	Risk §§§	NR	NR *No*	No *No*
Milano, 1988[30] France *Prospective case series*	5-fluorouracil (5-day continuous infusion)	None *HPLC*	26	Counts ¥	3/26 *(12)*	NR *No*	NR *No*
Stremetzne, 1999[31] Germany *RCT**	5-fluorouracil (5-day continuous) + FA	None *HPLC*	16	Risk §	0/16 *(0)*	NR *No*	NR *No*
Ychou, 1999[32] France *Prospective case series*	de Gramont (LV55-fluorouracil2)	tested 2 algorithms *HPLC*	38	Risk & Counts	Unclear	NR *No*	NR *No*
Ychou, 2003[33] France. *Prospective case series*	de Gramont (LV55-fluorouracil2)	Ychou, 1999[32] *HPLC*	53	Risk ¥¥	19/52 *(36.5)*	18.6 *No*	7 *No*
Yoshida, 1990[34] Japan. *Prospective case series*	5-fluorouracil	None *HPLC*	19	Risk§ (Total toxicities only)	10/19 *(53)*	NR *No*	NR *No*

LV = leukovorin = FA = folinic acid; inf = infusion; NR = not reported; HPLC = High Performance Liquid Chromatography. All studies included advanced / metastatic colorectal cancer patients; Kline stratified according stage II/III or stage IV (data applies for stage IV); * studies randomised patients to two different dose regimens of folinate; ¶ Grade 3 or deemed sufficiently serious by the physician to warrant a dose reduction and “designated as adverse effects”; § time of assessment unclear; §§ reported extensively but irregularities in numbers reported §§§ inconsistent grouping of toxicity grades; ¥ grouping grade I+II & III+IV; ¥¥ grouping Cutaneous and Haematological III+IV, Digestive and mucositis III+IV; HPLC = high performance liquid chromatography
