# Supplementary material for: Is monitoring of plasma 5-fluorouracil levels in metastatic / advanced colorectal cancer clinically effective? A systematic review
Source: BMC Cancer. 2016 Jul 25;16:523. doi: 10.1186/s12885-016-2581-x (PMC4960837; doi:10.1186/s12885-016-2581-x)
Supplement: Additional file 4: — Characteristics of studies testing if My5-FU is clinically equivalent to LC-MS/MS. (PDF 14 kb) [file 12885_2016_2581_MOESM4_ESM.pdf]

ADDITIONAL FILE 4. Characteristics of studies testing if My5-FU is clinically equivalent to LC-MS/MS

**Study**<br>***Sample N***<br>***Source***	**Range of plasma concentrations * (ng/mL)**	**Correlation**	**Automation of My5-FU assay**	**Bland-Altman Plot**		
				**Bias (95%CI)**	**Lower & upper limits of agreement**	**Largest outliers**
Buchel, 2013[38]<br>*247*<br>*GI cancers*	93 to 17881	R^2=0.99**<br>Slope 1.08 (95%CI 1.06 to 1.09)	Roche Cobas Integra 800 analyser	7.0% (5.5 to 8.5)	-18% 30% §§	-50% +95%§§
Beumer, 2009[15]<br>*156*<br>*Head and Neck and colorectal cancer*	93-1774	R^2=0.97<br>Slope 1.035<br>Intercept 10.9ng/mL	Olympus AU400 analyser	+ 23ng/mL (NR)	NR	-35% +52%§§
Makihara 2012[39]<br>*50*<br>*colorectal cancer§*	41-457	R^2=0.8471	None or NR	NR	NR	NR

**as measured by the index test; **Passing-Bablok regression*[52] ; *§ patients received prodrug; §§ read from plot ,capecitabine; NR = not reported;*
