# Supplementary material for: Is monitoring of plasma 5-fluorouracil levels in metastatic / advanced colorectal cancer clinically effective? A systematic review
Source: BMC Cancer. 2016 Jul 25;16:523. doi: 10.1186/s12885-016-2581-x (PMC4960837; doi:10.1186/s12885-016-2581-x)
Supplement: Additional file 5: — Dose adjustment algorithms based on plasma 5-fluorouracil measurement in colorectal cancer patients. (PDF 148 kb) [file 12885_2016_2581_MOESM5_ESM.pdf]

ADDITIONAL FILE 5. Dose adjustment algorithms based on plasma 5-fluorouracil measurement in colorectal cancer patients

**Gamelin et al. (1996)**[24] : 8 hour 5-fluorouracil infusion regimen + FA; initial dose 1,500 mg/m^2						
Plasma 5-fluorouracil (µg/L)	**AUC mg*H/L**	**Dose change § (%)**	***Plasma 5-fluorouracil (µg/L)***	**AUC mg*H/L**	**Dose change§ (%)**	**In case of toxicity**
<500	<4	+50%	*1800-2000*	14.4-16	+5%	Grade 2 toxicity: 200mg dose decrease Grade 3 toxicity: 1 week break then 300mg dose decrease
500-1000	4-8	+40%	***2000-3000***	**16-24**	**None**	
1000-1300	8-10.4	+30%	*3000-3200*	24-25.6	-5%	
1300-1500	10.4-12	+20%	*3200-3500*	25.6-28	-10%	
1500-1800	12-14.4	+10%	*>3500*	>28	-30%	
§ percentage change from previous cycle dose; H = hours of 5-fluorouracil infusion						

**Kaldate et al. (2012)**[40] : 46 hour 5-fluorouracil infusion + oxaloplatin + FA; initial dose 2,400 to 3,000 mg/m^2 after a 400 mg/m^2 bolus					
Plasma 5-fluorouracil (µg/L) ¥	**AUC §§ mg*H/L**	**Change in dose (mg/m^2)**	***Plasma 5-fluorouracil (µg/L) ¥***	**AUC §§ mg*H/L**	**Change in dose (mg/m^2)**
174-217	8–10	↑727			
239-283	11–13	↑582	*672-717*	31–33	↓291
304-348	14–16	↑436	*739-783*	34–36	↓436
370-413	17–19	↑291	*804-848*	37–39	↓582
435-652	**20–30**	**None**	*870*	≥40	↓727
¥ Calculated assuming infusion of 46 hours; §§ area under the curve of previous cycle; H = hours of 5-fluorouracil infusion					

**Ychou et al. (1999)**[32] 400 mg/ m^2 bolus 5-fluorouracil; then a 22 hour infusion of 600 mg/ m^2 5-fluorouracil repeated the next day; +FA	
**AUC §§§ mg*H/L*m^2**	**Dose increase § (%)**
≤5	150%
5<AUC≤10	100%
10<AUC≤15	50%
15<AUC≤20	25%
>20	No increase
**§§§** note differently reported pharmacokinetic units and calculation for area under the curve relative to the other algorithms	
