# Supplementary material for: Is monitoring of plasma 5-fluorouracil levels in metastatic / advanced colorectal cancer clinically effective? A systematic review
Source: BMC Cancer. 2016 Jul 25;16:523. doi: 10.1186/s12885-016-2581-x (PMC4960837; doi:10.1186/s12885-016-2581-x)
Supplement: Additional file 6: — Consistency of [A] overall survival and [B] progression free survival in body surface area guided dose arms of studies. (PDF 124 kb) [file 12885_2016_2581_MOESM6_ESM.pdf]

ADDITIONAL FILE 6. Consistency of [A] overall survival and [B] progression free survival in body surface area guided dose arms of studies.

[A] Overall survival

Parmacokinetic studies may appear to show an advantage for pharmacokinetic regimens relative to BSA regimens because of poor performance in the BSA arm of comparative studies. Figure 1 examines overall survival in BSA regimens in which either 5-fluorouracil + FA and for FOLFOX 6 treatments were used. The left column shows reconstructed Kaplan-Meier plots for 5-fluorouracil + FA and for FOLFOX 6 regimens respectively and the right column shows Weibull distribution models used to estimate mean and median survival times.

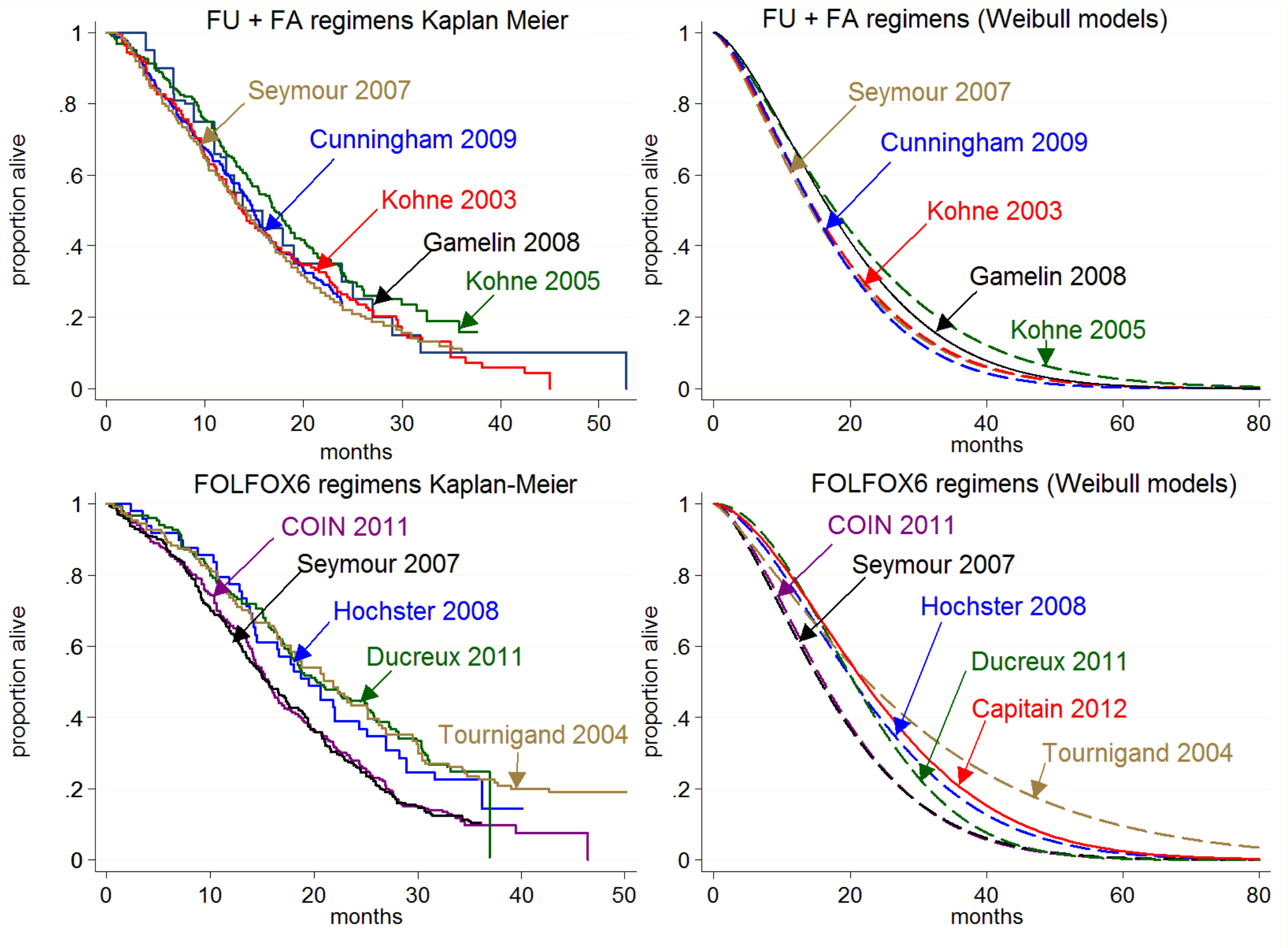

Figure 1. Consistency of reported overall survival in body surface area studies using 5-fluorouracil + FA and FOLFOX 6 regimens.

Note [a] in the two comparative studies (Gamelin 2008 and Capitain 2012) body surface area overall survival is at least as good as that in other studies; [b] the two UK studies (COIN 2011 and Seymour 2007) report very similar overall survival but somewhat poorer than seen in the other studies. Capitain 2012 (red solid line) is modelled using reported median of 22 months. Patients in Tournigand received second line FOLFIRI on progression.

[B] Progression free survival.

Figure 2 examines progression free survival in BSA regimens in which either 5-fluorouracil + FA or FOLFOX 6 treatments were used. The left column shows reconstructed Kaplan-Meier plots for 5-fluorouracil + FA and for FOLFOX 6 regimens and the right column shows Weibull distribution models used to estimate mean and median survival times. Progression free survival for the PKA arms of available studies is also shown.

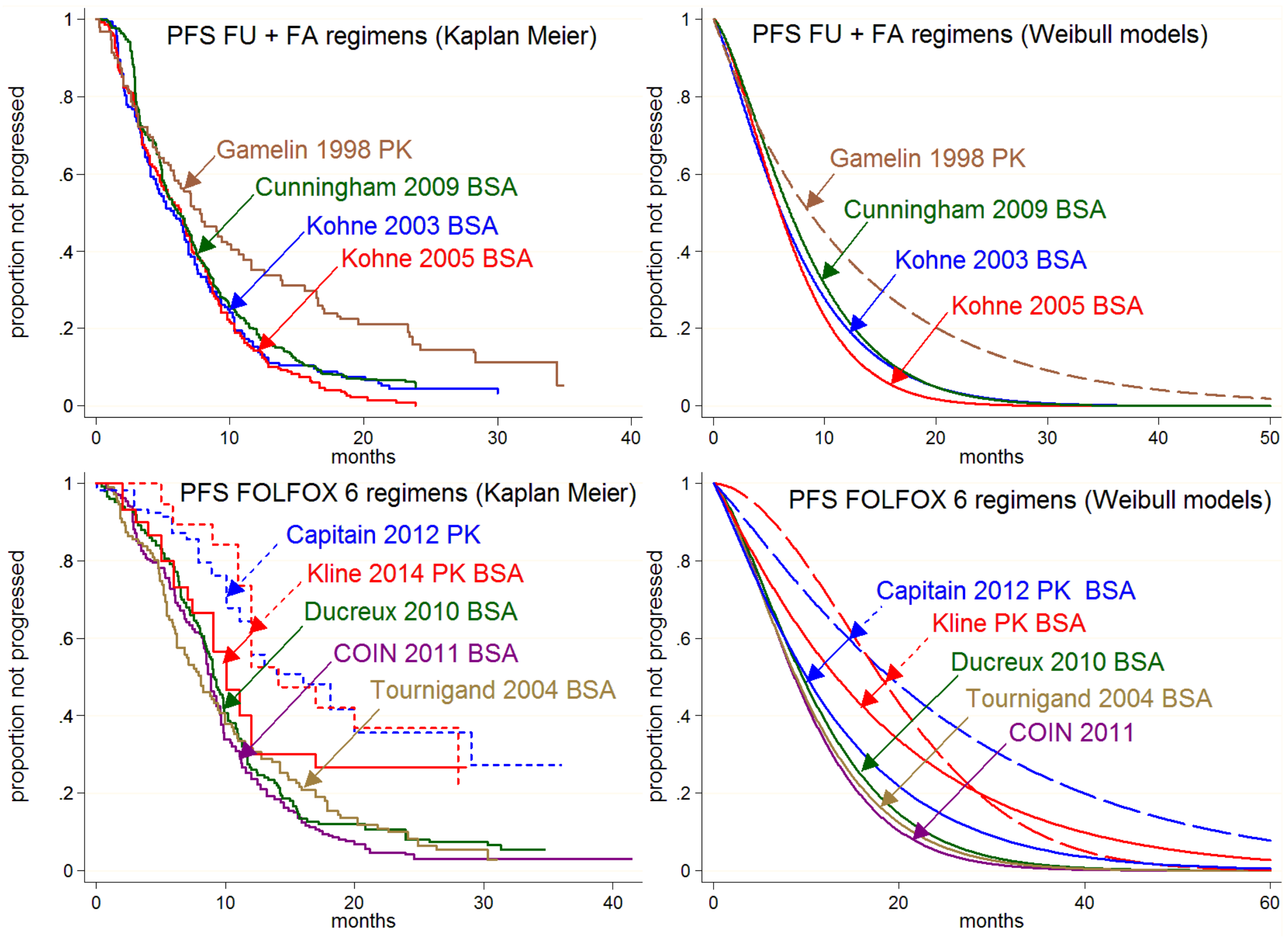

Figure 2 Consistency of reported progression-free survival in pharmacokinetic and body surface area studies using 5-fluorouracil + FA and FOLFOX 6 regimens
Note: Gamelin 1998 was a single arm PKA study, Capitain 2012 was a comparative study that reported a KM plot for the PKA arm but only median PFS for the BSA arm, Kline was a comparative study that reported a KM plot for both arms. Note body surface area progression-free survival is at least as good in the Kline and Capitain as in other studies; Capitain 2012 body surface area progression-free survival (solid line) is modelled using reported median of 10 months.
