# Supplementary material for: Is monitoring of plasma 5-fluorouracil levels in metastatic / advanced colorectal cancer clinically effective? A systematic review
Source: BMC Cancer. 2016 Jul 25;16:523. doi: 10.1186/s12885-016-2581-x (PMC4960837; doi:10.1186/s12885-016-2581-x)
Supplement: Additional file 7: — Modelled median and mean overall survival in pharmacokinetic and body surface area arms in studies of 5-fluorouracil + FA and FOLFOX6. (PDF 105 kb) [file 12885_2016_2581_MOESM7_ESM.pdf]

ADDITIONAL FILE 7.

Modelled median and mean overall survival in pharmacokinetic and body surface area arms in studies of 5-fluorouracil+FA and FOLFOX6

Table 1 Overall survival time (months)

	Body surface area overall survival 5-fluorouracil + FA			Pharmacokinetic overall survival 5-fluorouracil + FA	
Study	Weibull median	Weibull mean (95% CI)	Study	Weibull median	Weibull mean
			Gamelin 1998[25]	20.8	25.44 (21.80-29.44)
			Capitain 2008[20]	18.4	23.44 (18.70-29.33)
Gamelin 2008[35]	17.0	19.64 (16.82-22.77)	Gamelin 2008[35]	20.8	22.61 (19.65-25.85)
Cunningham 2009[46]	14.7	16.89 (16.82-22.77)			
Kohne 2005[44]	17.8	21.28 (18.72-24.27)			
Kohne 2003[43]	14.9	17.57 (15.20-22.36)			
Seymour 2007[45]	14.3	17.12 (15.95-18.39)			
Studies combined	15. 2	18. 0	Studies combined	20.2	24.1
	Body surface area overall survival FOLFOX 6			Pharmacokinetic overall survival FOLFOX 6	
Study	Weibull median	Weibull mean	Study	Weibull median	Weibull mean
COIN 2011[50]	16.1	18.32 (16.67-20.09)			
Seymour 2007[45]	15.7	18.10 (16.48-19.89)			
Hochster 2008[49]	20.5	22.98 (18.20-29.10)			
Ducreux 2011[48]	20.5	21.93 (19.73-24.27)			
Tournigand 2004[47]	22.6	28.19 (23.10-34.80)			
Capitain 2012[36]	22	24.5	Capitain 2012[36]	30.3	33.73 (29.21-38.93)

Table 2. Progression free survival time (PFS) (months)

	Body surface area PFS 5-fluorouracil + FA			Pharmacokinetic PFS 5-fluorouracil + FA	
Study	Weibull median	Weibull mean (95%CI)	Study	Weibull median (months)	Weibull mean (months)
Kohne 2003[43]	6.1	7.65 (6.58-8.90)			
Kohne 2005[44]	6.0	6.97 (6.24-7.77)			
Cunningham 2009[46]	6.9	8.21 (7.49-8.98)			
			Gamelin 1998[25]	8.7	12.54 (10.35-15.23)
	Body surface area PFS FOLFOX 6			Pharmacokinetic PFS FOLFOX6	
	Weibull median (months)	Weibull mean (months)		Weibull median (months)	Weibull mean (months)
COIN 2011[50]	8.7	10.23 (9.24-11.29)			
Ducreux 2011[48]	9.6	11.41 (9.90-13.14)			
Tournigand 2004[47]	8.9	10.66 (9.01-12.59)			
Kline 2014[37]	13.2	17.91 (11.40-31.48)	Kline 2014[37]	18.0	19.57 (13.49-29.06)
Capitain 2012[20]	10.0	13.2	Capitain 2012[20]	19.0	25.07 (20.04-32.18)
