# Supplementary material for: Is monitoring of plasma 5-fluorouracil levels in metastatic / advanced colorectal cancer clinically effective? A systematic review
Source: BMC Cancer. 2016 Jul 25;16:523. doi: 10.1186/s12885-016-2581-x (PMC4960837; doi:10.1186/s12885-016-2581-x)

ADDITIONAL FILE 8.

Parametric models of overall survival and progression free survival in 5-fluorouracil+ folinate and FOLFOX6 regimens. Red lines = Weibull models, blue lines = lognormal models

Figure 1. Weibull and loglogistic fits to overall survival in FOLFOX 6 regimens.

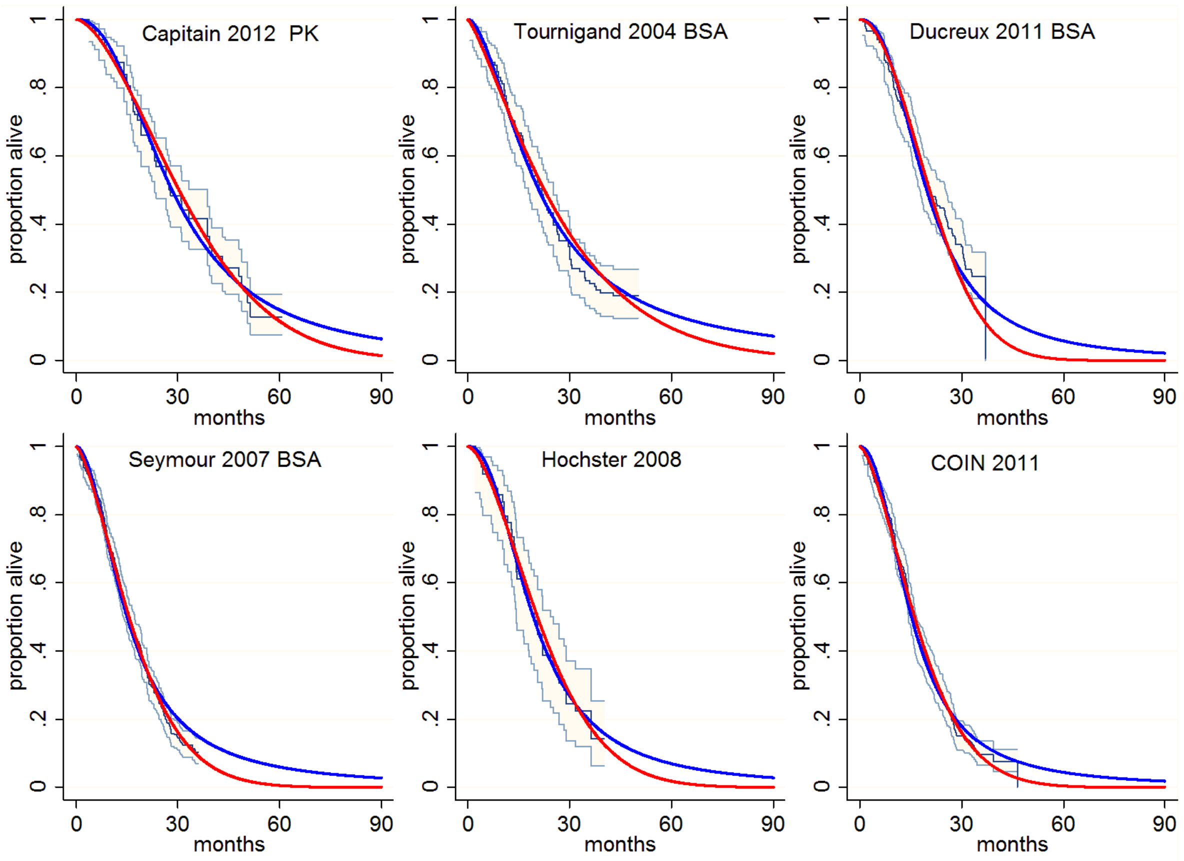

Figure 2. Weibull and loglogistic fits to overall survival in 5-fluorouracil + FA regimens.

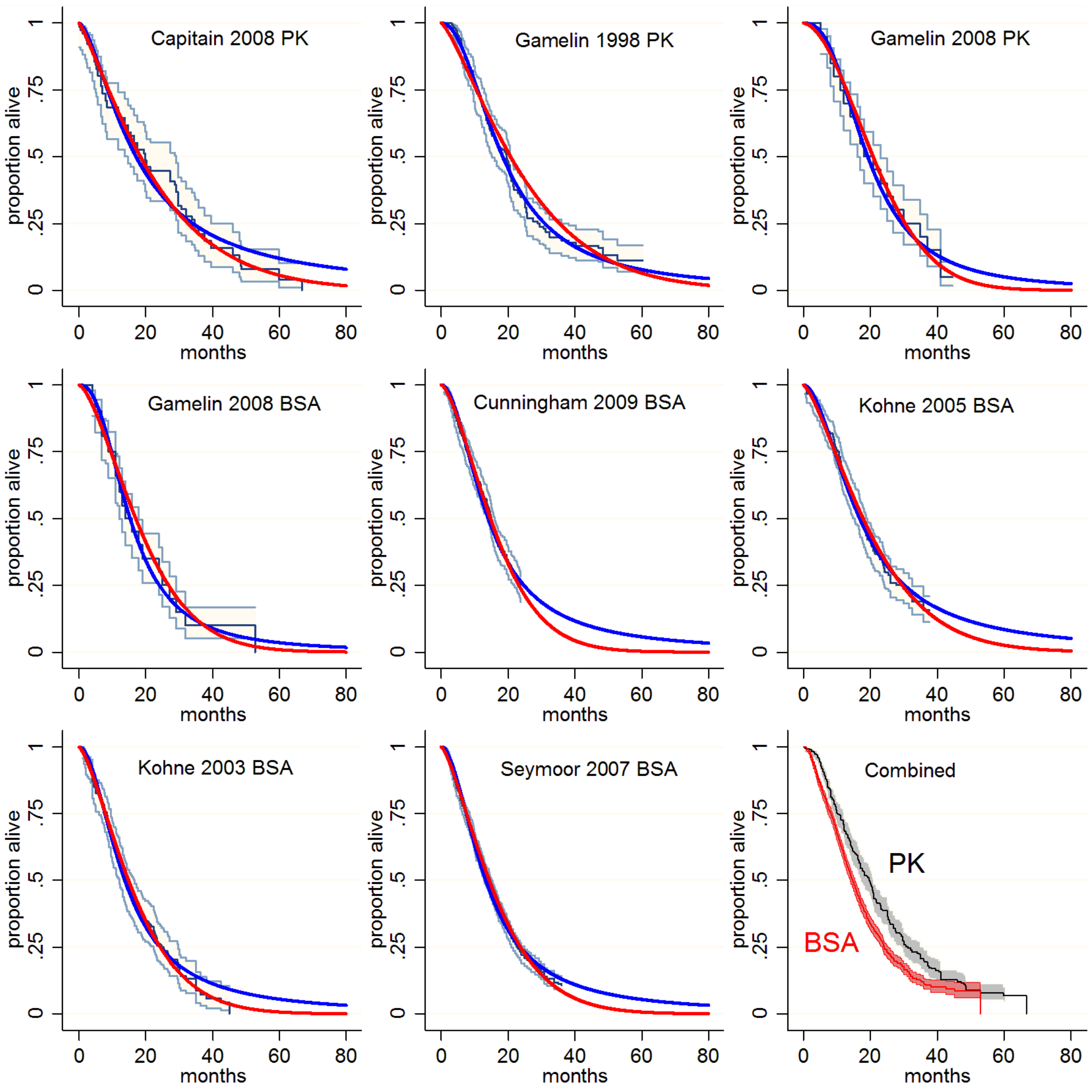

Figure 3. Weibull and loglogistic fits to progression-free survival in FOLFOX 6 regimens.

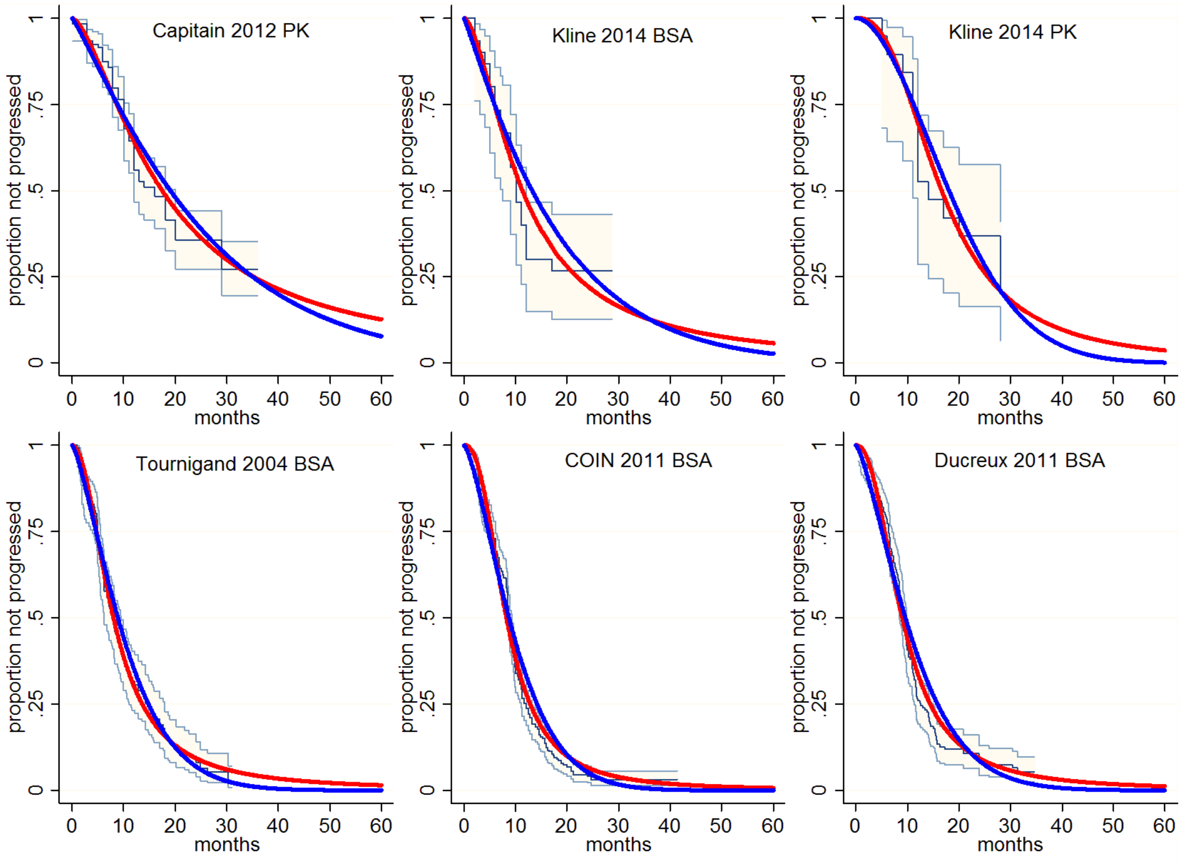

Figure 4. Weibull and loglogistic fits to progression-free survival in 5-fluorouracil + FA regimens.

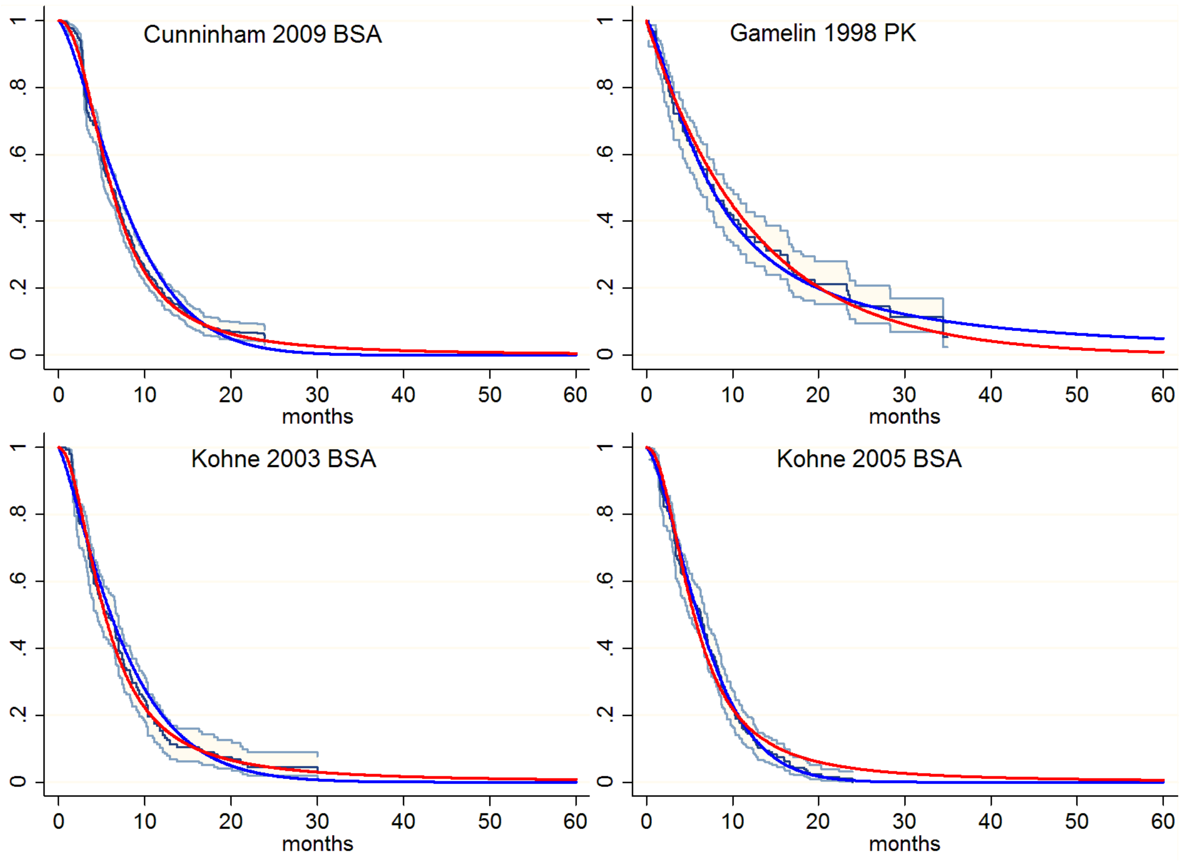

Supplement: Additional file 8: — Parametric models of overall survival and progression free survival in 5-fluorouracil + folinate and FOLFOX6 regimens. (PDF 182 kb) [file 12885_2016_2581_MOESM8_ESM.pdf]
