# Supplementary material for: Is monitoring of plasma 5-fluorouracil levels in metastatic / advanced colorectal cancer clinically effective? A systematic review
Source: BMC Cancer. 2016 Jul 25;16:523. doi: 10.1186/s12885-016-2581-x (PMC4960837; doi:10.1186/s12885-016-2581-x)

ADDITIONAL FILE 9.

Relative risk of adverse events in the RCT of Gamelin (PK versus BSA dose adjustment strategies)

Adverse event	RR (95% CI)	Events, Treatment	Events, Control
1			
WHO_grade_III (cardiac toxicity)	1.07 (0.07, 16.80)	1/90	1/96
WHO_grade_II (cardiac toxicity)	3.20 (0.13, 77.50)	1/90	0/96
WHO_grade_I (cardiac toxicity)	3.20 (0.13, 77.50)	1/90	0/96
WHO_grade_IV (cardiac toxicity)	(Excluded)	0/90	0/96
.			
2			
WHO_grade_IV (mucocitis)	1.07 (0.15, 7.41)	2/90	2/96
WHO_grade_III (mucocitis)	1.07 (0.07, 16.80)	1/90	1/96
WHO_grade_II (mucocitis)	1.07 (0.07, 16.80)	1/90	1/96
WHO_grade_I (mucocitis)	1.07 (0.07, 16.80)	1/90	1/96
.			
3			
WHO_grade_IV (leukopenia)	0.36 (0.01, 8.61)	0/90	1/96
WHO_grade_III (leukopenia)	0.36 (0.01, 8.61)	0/90	1/96
WHO_grade_II (leukopenia)	0.21 (0.01, 4.38)	0/90	2/96
WHO_grade_I (leukopenia)	(Excluded)	0/90	0/96
.			
4			
WHO_grade_IV (diarrhoea)	0.15 (0.01, 2.91)	0/90	3/96
WHO_grade_III (diarrhoea)	0.30 (0.10, 0.89)	4/90	14/96
WHO_grade_II (diarrhoea)	0.13 (0.04, 0.41)	3/90	25/96
WHO_grade_I (diarrhoea)	0.74 (0.33, 1.64)	9/90	13/96
.			
5			
WHO_grade_IV (hand & foot)	3.20 (0.13, 77.50)	1/90	0/96
WHO_grade_III (hand & foot)	1.78 (0.67, 4.69)	10/90	6/96
WHO_grade_II (hand & foot)	1.07 (0.62, 1.85)	20/90	20/96
WHO_grade_I (hand & foot)	2.06 (1.19, 3.59)	29/90	15/96
.			
6			
WHO_grade_II (conjunctivitis)	2.67 (0.53, 13.40)	5/90	2/96
WHO_grade_I (conjunctivitis)	0.53 (0.26, 1.08)	10/90	20/96
WHO_grade_IV (conjunctivitis)	(Excluded)	0/90	0/96
WHO_grade_III (conjunctivitis)	(Excluded)	0/90	0/96
.			

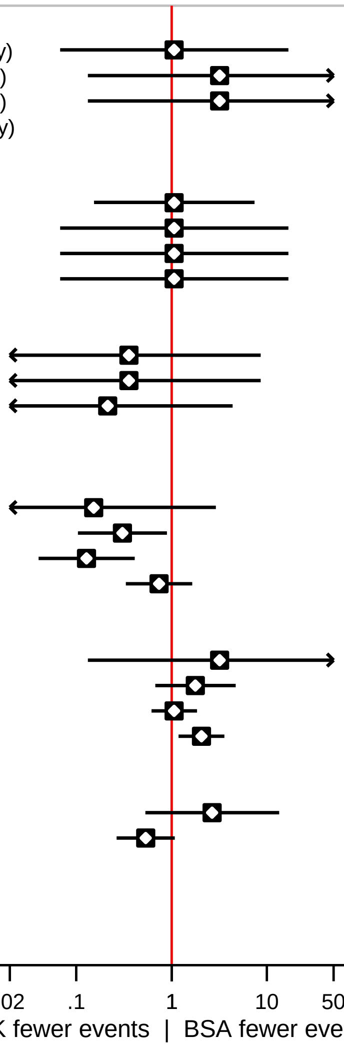

Supplement: Additional file 9: — Relative risk of adverse events in the RCT of Gamelin (PK versus BSA dose adjustment strategies). (PDF 8 kb) [file 12885_2016_2581_MOESM9_ESM.pdf]
